# Electrodynamic Interference and Induced Polarization in Nanoparticle-Based Optical Matter Arrays

**DOI:** 10.1021/acs.jpcc.3c08459

**Published:** 2024-04-26

**Authors:** Curtis Peterson, John Parker, Emmanuel Valenton, Yuval Yifat, Shiqi Chen, Stuart A. Rice, Norbert F. Scherer

**Affiliations:** † Department of Chemistry, The University of Chicago, Chicago, Illinois 60637, United States; ‡ James Franck Institute, The University of Chicago, Chicago, Illinois 60637, United States; § Department of Physics, The University of Chicago, Chicago, Illinois 60637, United States

## Abstract

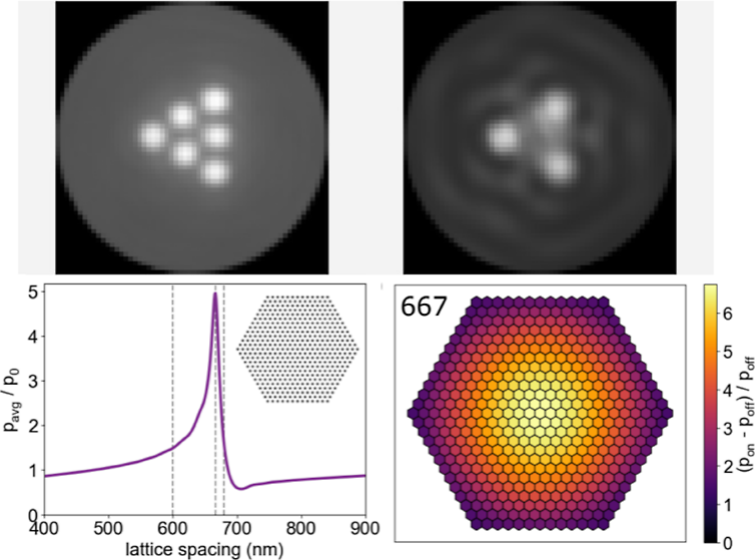

Optical matter (OM) arrays are self-organizing, ordered arrangements of nanometer- to micrometer-size particles, where interparticle forces are mediated by incident and scattered coherent light. The structures that form and their dynamics depend on the properties (e.g., material, size) of the constituent particles, as well as the incident and scattered light. While significant progress has been made toward understanding how the OM arrays are affected by the phase, polarization, and intensity profile of the incident light, the polarization induced in the particles and the light scattered by OM arrays have received less attention. In this paper, we establish the roles of electrodynamic interference, many-body coupling, and induced-polarization concomitant with the coherent light scattered by OM arrays. Experiments and simulations together demonstrate that the spatial profile and directionality of coherent light scattered by OM arrays in the far field are primarily influenced by interference, while electrodynamic coupling (interactions) and the associated polarization induced in the nanoparticle constituents have a quantitative wavelength-dependent effect on the total amount of light scattered by the arrays. Furthermore, the electrodynamic coupling in silver nanoparticle OM arrays is significantly enhanced by constructive interference and increases superextensively with the number of particles in the array. Particle size, and hence polarizability, also has a significant effect on the strength of the coupling. Finally, we simulate larger hexagonal OM arrays of Ag nanoparticles to demonstrate that the electrodynamic coupling and scattering enhancement observed in small OM arrays develop into surface lattice resonances observed in the infinite array limit. Our work provides insights for designing OM arrays to tune many-body forces and the coherent light that they scatter.

## Introduction

In general, two or more particles simultaneously present in optical traps interact electrodynamically with one another, and these interactions produce optical binding forces.^1^ As a result, the particles tend to self-organize into ordered optical matter (OM) arrays with preferred interparticle separations at (near) integer multiples of the incident laser wavelength.^1−7^ The optical binding forces arise from the interaction between the polarization induced in each particle by the light incident and scattered from other particles.^4,8^ OM arrays are open, nonequilibrium systems because the coherent light source that mediates the optical binding forces also provides a constant flux of electromagnetic energy through the system.^9^ Conversion or redirection of the momentum from the incident laser light makes possible phenomena such as nonreciprocal forces,^9−11^ negative optical torque,^12−16^ OM machines,^16−18^ and nanoscale light sails.^19^ Therefore, a full description of an OM array requires knowing the detailed properties of both the incident and scattered electromagnetic fields, in addition to the positions, sizes, shapes, and composition of each particle.

While there has been steady progress toward understanding how tailoring the phase and intensity profiles of the incident fields can affect the dynamics and structures formed by optically trapped plasmonic nanoparticles,^6,20−26^ the characteristics of coherent light scattered by OM arrays are an area of current research.^16,27−29^ The periodic wavelength-scale structures of OM arrays^30^ suggest that electromagnetic interference plays an important role in the properties of the light they scatter. In addition, the large scattering cross sections of the plasmonic nanoparticles that OM arrays are often comprised of^5,7^ suggest that electrodynamic coupling (i.e., interactions) may also be important, leading to new forces and collective or many-body properties and behaviors.^16,17,31−33^ However, the respective roles of electromagnetic interference and electrodynamic coupling with respect to the coherent light scattered by OM arrays and their interdependency have received little attention.

By electrodynamic coupling, we mean the polarization induced in one particle due to light scattered by another particle, which can be categorized into two regimes.^34−36^ In near-field coupling, the interaction between particles with separations much smaller than the wavelength of light is treated as quasi-static.^37,38^ When objects in coherent electromagnetic fields are separated by distances on the scale of that wavelength, their interactions are described as far-field coupling and occur through scattered radiation. Both types of coupling modify the induced polarization of a particle in the array due to light scattered by other nearby particles. In large arrays, where far-field coupling dramatically affects each particle’s induced polarization, the interaction between particles is frequently treated analogously to methods in solid-state physics, i.e., with approaches that invoke the periodicity of the array.^39−42^ It is important to note that coupling and the associated induced polarizations give rise to the many-body nature of OM arrays.^16,33^ Near-field coupling is significantly stronger than far-field coupling and is often studied on a pairwise basis.^37,38^ Far-field coupling is usually studied in the limit of very large arrays, although some research has examined finite-size effects.^43^ The approaches typically used in the near- and far-field coupling regimes are not suitable for describing coupling in small OM arrays: a quasi-static approach is inappropriate because retardation is significant over the wavelength-scale distances characteristic of OM arrays, and the edges and boundaries of finite-size arrays preclude momentum space representations.

In this paper, we show that OM arrays exist in a regime where interference determines the spatial profile of the light scattered by the array, but coupling (and multiple scattering) enhances the polarization induced in the array’s constituent material elements (e.g., metal or dielectric nanoparticles). These properties have visual manifestations. We show that the images obtained with coherent backscattered light (termed coherent imaging) from OM arrays contrast strongly with those obtained with incoherent light. While diffraction (i.e., fringe) patterns visible in the coherent images characterize the electric field intensity surrounding the OM arrays, the more striking finding is the replacement of particle-centric images obtained with incoherent light illumination with images where the intensity is shifted between particles when visualized with scattered coherent light. We investigate the directional scattering of coherent light over polar angles by performing generalized multiparticle Mie theory (GMMT) calculations of ordered OM arrays with 1–7 particles.^16,44^ In contrast to the largely dipolar scattering of a single particle,^45,46^ the light scattered from ordered OM arrays develops a lobed structure with maxima in specific sideways, forward, and backward directions. This scattering can also be described in terms of collective modes that arise from multiparticle coupling.^16,43^

We find that the total scattering of small OM arrays at the trapping laser wavelength grows superextensively (i.e., faster than linear) when nanoparticles are added to the array. The superextensive growth of the scattering is the result of “all with all” electrodynamic coupling. We quantify the strength of electrodynamic coupling in OM arrays at the trapping laser wavelength by calculating the ratio of the total electric field intensity to the incident intensity at a vacant site in the array, finding that the contribution from neighboring particles becomes significant even for small (1–6 neighboring particles) OM arrays. The measured scattering and local density of states (LDOS) enhancement for a range of wavelengths shows that both increase near the trapping laser wavelength as more particles are added to the OM array, and a collective resonance develops at the expense of the single-particle Mie resonance scattering from individual particles.^43^ We also show simulated and experimental backscattered spectra that demonstrate the scattering enhancement of a coherent light source by OM arrays. Finally, we extend our investigation to larger hexagonal arrays to show the connection between OM arrays of plasmonic nanoparticles and surface lattice resonances (SLR’s; also known as lattice plasmons).^35,39−41,47,48^ Specifically, the sharp resonances that yield scattering enhancement in large regular arrays of nanoparticles occur concurrently with enhancement of the induced polarization similar to that seen in small OM arrays.

We interpret our results in the context of analytical theory within the point-dipole approximation and show that electrodynamic coupling in OM arrays is strengthened by constructive interference. Specifically, the large scattering cross sections of the plasmonic particles often used in OM experiments, the emergent periodic structures that self-organize, and the wavelength-scale separations between the particles all play important roles. Our work demonstrates that collective excitations in OM arrays are equivalent to SLR’s in the small lattice-size regime.^43^

## Experimental Setup

**Figure 1:**
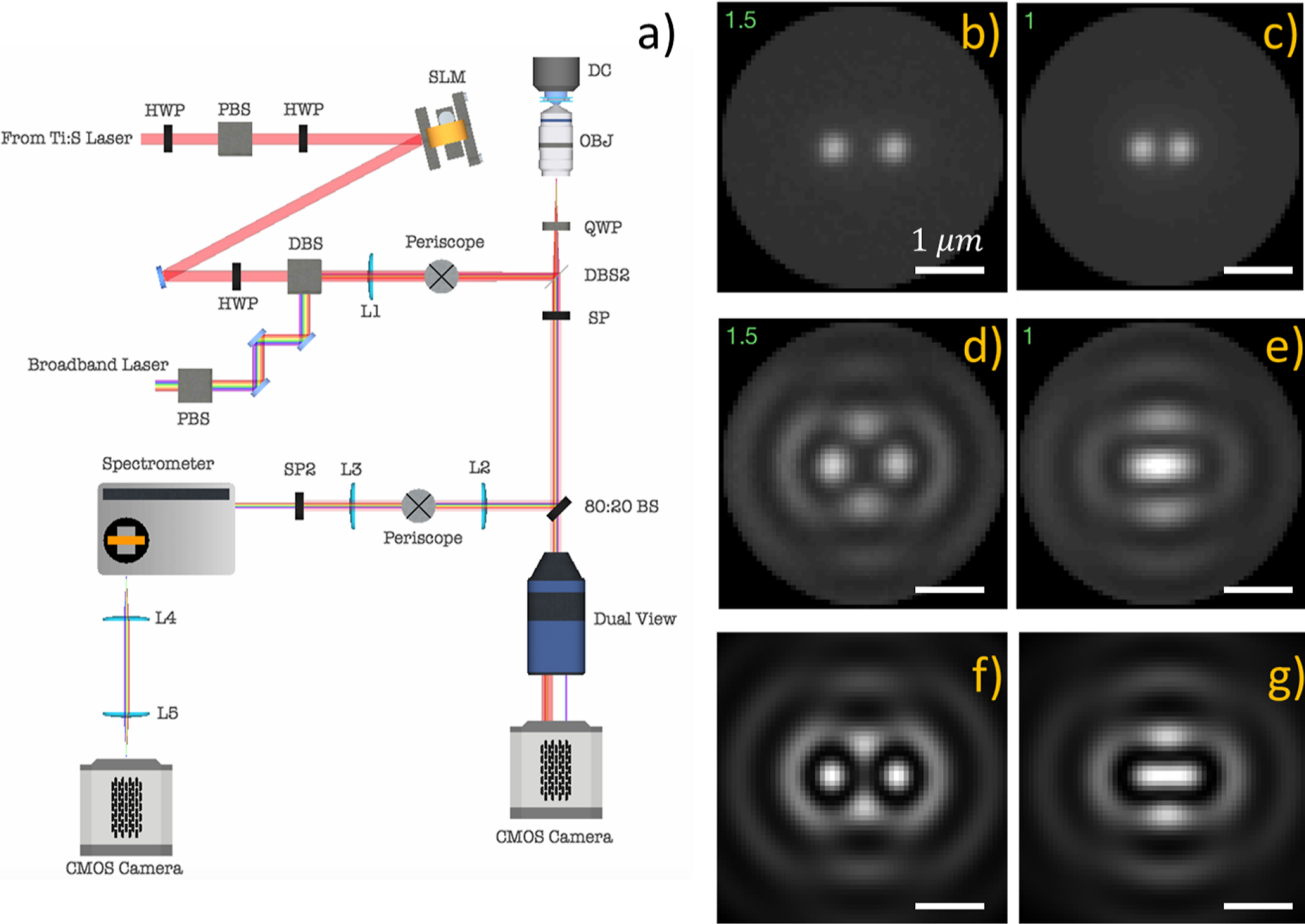
Optical trapping setup and averaged images of dimers. (a) Optical trapping setup with simultaneous video microscopy and backscattered spectral measurements. HWP—half wave plate; QWP—quarter wave plate; SLM—spatial light modulator; DBS—dichroic beam splitter; and PBS—polarizing beam splitter; and SP—notch filter. (b,c) Incoherent light darkfield (NA = 1.27) images of a NP dimer at separations of 1.5λ (b) and λ (c). (d,e) Coherent light-backscattered (NA = 1.27) images of the NP dimer at separations of 1.5λ (d) and λ (e). (f,g) Simulated coherent light-backscattered (NA = 1.00) images of NP dimer at separations of 1.5λ (f) and λ (g). See Supporting Videos 2 and 3 for a sequence of images for different interparticle separations obtained with incoherent and coherent light.

Our experiments were conducted with a single-beam optical tweezers in an inverted microscope, as described previously.^49^ A schematic of the experimental setup is shown in Figure 1. A dilute aqueous solution of PVP-coated 150 nm Ag nanoparticles was placed inside a sample chamber made from an adhesive spacer sandwiched between two glass coverslips. A continuous wave Ti-sapphire laser was slowly diverging at the back aperture of a 60× microscope objective (Nikon SAPO 60× water; numerical aperture (NA) = 1.27), creating a converging beam at the plane of the array of Ag nanoparticles. The radiation pressure from the beam was balanced by electrostatic repulsion of the PVP molecules on the Ag nanoparticles by the charged upper glass surface of the sample chamber, causing a small number of nanoparticles to be trapped close to the top glass surface. The focus of the optical trapping beam was adjusted with a spatial light modulator (SLM; Meadowlark) to create an inwardly directed phase gradient at the trapping plane that increased the confinement of the nanoparticles.^16,21^ The trapping laser was circularly polarized in all experiments and calculations.

To image the coherent backscattered light, we employed a 2-channel configuration where one channel allowed detection of 470 nm incoherent LED dark-field illumination, and the other channel filtered out the LED light and allowed detection of the backscattered laser light but with significant attenuation (OD = 5). The two channels form spatially separated images on the same CMOS detector (Andor Neo). The simultaneous measurements are necessary because the particle positions are not obvious from the images of backscattered coherent light, as can be seen in Figure 1b–g. No additional field stops or aperture stops were introduced to the optical path, so the nominal NA is that of the objective.

To experimentally determine the wavelength-dependent scattering enhancement in OM arrays, we measured backscattered spectra using a spatially coherent broadband source. A backscattering geometry was chosen for excitation and detection, where the direction of propagation is normal to the plane of the array so that each particle in the array is excited with the same phase. Although we anticipate a scattering enhancement at wavelengths near that of the trapping laser (because it defines the characteristic optical binding distance), the trapping laser wavelength needs to be filtered out because it is much more intense than the spatially coherent broadband source. We employed a pulsed supercontinuum fiber laser (Fianium WL400-4-PP), operating at maximum power with a 5.00 MHz pulse repetition rate, coupled to a computer-controlled variable interference filter (Fianium SuperChrome) set to its maximum bandwidth. As shown in Figure 1a, the broadband beam was directed to travel collinear with the trapping laser into the optical trap, and the backscattered light was sent through a DBS and notch filter (Semrock StopLine NF03—25) to remove the trapping laser intensity from the backscattered light. 20% of the light was directed toward a CMOS array detector (Andor Neo) for imaging, and the remaining 80% of the light was directed toward a spectrograph (Andor Shamrock SR-193i-B1-SIL). A pair of relay lenses (Thorlabs AC508-100-B-ML) with a focal length *f* = 100 mm were then used to bring the resulting spectrum from the spectrograph to a second CMOS array detector (Andor NEO). The imaging and spectral CMOS detectors were synchronized so that the spectral measurement was acquired at the same frame rate as the imaging. Both detectors were started, and 1000 images and spectra were acquired at 160 fps once an OM array had formed. The spectra were classified by (i) specific numbers of nanoparticles and (ii) as arising from ordered vs disordered arrays based on the fitting error (i.e., deviations of the particle positions from the lattice), resulting from real-space lattice fitting of the OM arrays in each frame.

### Coherent Imaging of OM Arrays

The optically trapped 150 nm diameter Ag nanoparticles in our experiments rotate, translate, and dynamically reconfigure in the aqueous solution due to the thermal energy of the bath (i.e., undergo Brownian motion).^16,27,49^ Therefore, dark-field microscopy videos (e.g., see Supporting Video 1) typically show particle arrays with fluctuating configurations where the probability of each specific configuration depends on the interparticle forces. Particle separations with integer multiples of the trapping wavelength in the solvent medium λ = λ_laser_/*n*, where *n* is the index of refraction, are favored due to optical binding.^1−5^

The individual images containing two randomly fluctuating particles in the optical trap were processed by the following protocol: (i) the two Ag nanoparticles were tracked in the images obtained with incoherent light using Mosaic (ImageJ); (ii) the particles were centered with respect to their “center of mass” and rotated with respect to the orientation of the pair; (iii) the oriented images were averaged in bins conditioned on interparticle separation to dramatically improve the signal-to-noise ratio of the images. See the Supporting Information for further details; see Video 1 for the raw data and Videos 2 and 3 for averaged and aligned videos measured with incoherent and coherent light, respectively.

Figure 1b,c shows averaged dark-field images measured with incoherent light where the pair of particles is separated by 1.5λ and λ, respectively. The images show that the incoherent light scattered from each of the particles is manifested as well-defined Gaussian spots regardless of interparticle distance to separations as small as 300 nm. Figure 1d,e shows averaged images measured with coherent light for the same separations. The images for particles separated by *r* = 1.5λ show two distinct spots, ostensibly near the particle locations, and a pattern of interference fringes around the dimer with two brighter spots on the perpendicular bisector between the particles. The image for *r* = λ shows a single elongated spot between the particle locations that is reminiscent of σ-bonding orbitals in diatomic molecules.^50^ The pattern of interference fringes also changes at *r* = λ compared to *r* = 1.5λ, with the first ring of fringes becoming ellipsoidal.

We performed GMMT calculations to generate simulated images for the particle configurations shown in Figure 1b–e (λ = 800 nm).^16,44,51−53^ GMMT is based on a generalization of single particle Mie theory^54^ to that of multiple particles illuminated by an arbitrary source using the translation theorems of the vector spherical harmonic wave functions.^51,55^ GMMT is particularly useful for OM systems because subwavelength particles largely only emit dipolar and quadrupolar scattering modes. Furthermore, GMMT accounts for all possible interactions between these modes in each particle. GMMT can be used to calculate the forces and torques on the surface of every particle. We developed an open source software, MiePy, was developed to efficiently implement GMMT in an easy-to-use and flexible Python library.^44^

The simulated images closely match each of our experimental results measured with coherent light scattered from the OM arrays when the simulated NA is set to 1.00. Fresnel reflection losses at high NA inside the objective may reduce the effective NA of the experimental image. Also, the particle images may be displaced from their true positions due to the spin-to-orbit angular momentum conversion of scattered light and the associated tilt of the scattered wavefront and shifting of the particle images in the transverse plane.^56^ Our imaging may capture an aspect of this displacement that blurs the averaged images. The image-averaging procedure also causes blurring. Therefore, despite the quoted experimental and simulated NA (1.27 vs 1.0, respectively), Figure 1 demonstrates that the image of a pair of nanoparticles illuminated by coherent light depends on the distance between them.

**Figure 2:**
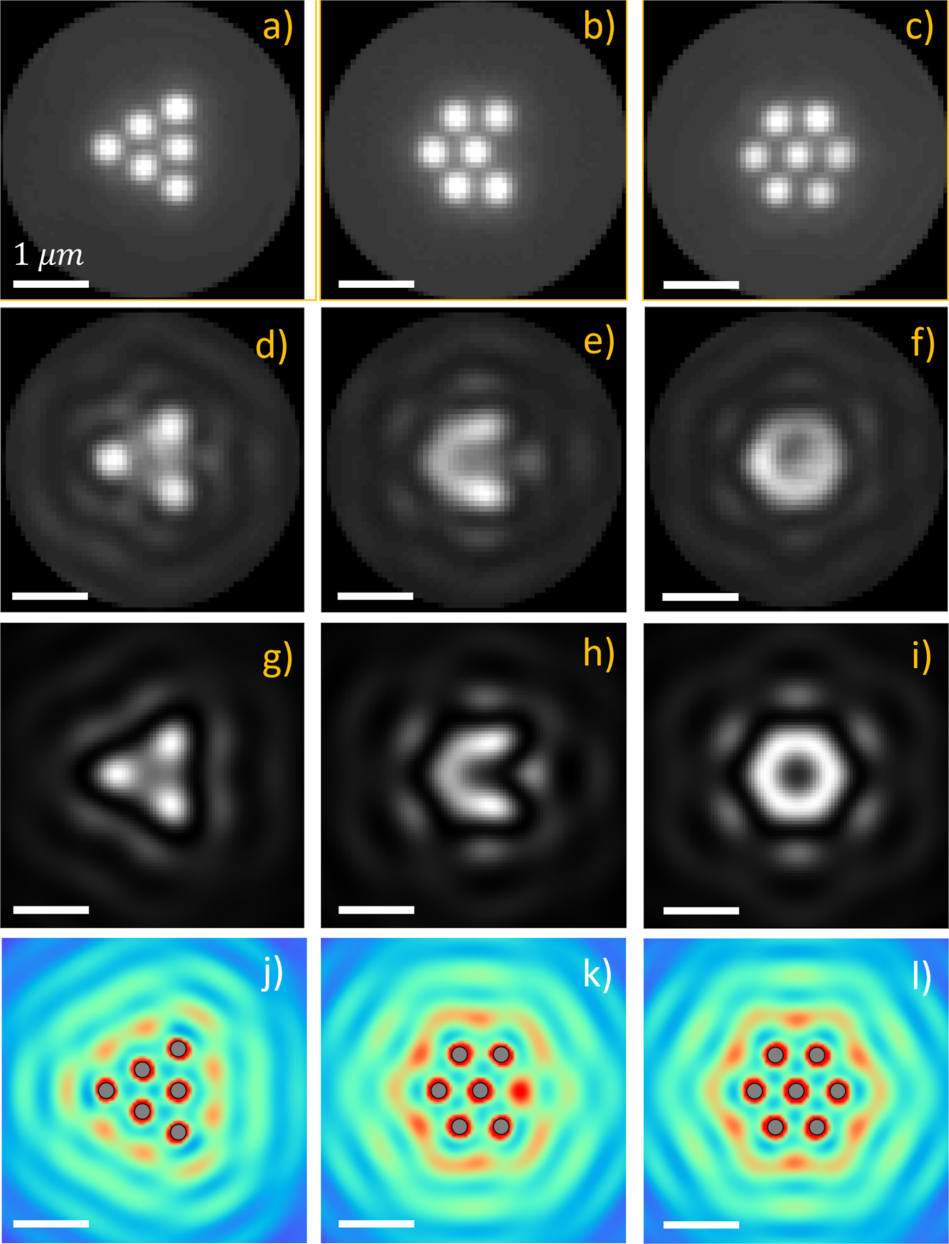
OM arrays imaged with incoherent and coherent light and comparison to the simulated electric field intensity. (a–c) Experimental incoherent backscattered images of OM arrays with 6 (a,b) and 7 (c) particles. (d–f) Experimental coherent backscattered images of OM arrays with 6 (d,e) and 7 (f) particles. (g–i) Simulated coherent backscattered images each of the three OM arrays as panels (a–c), respectively. (j–l) Simulated electric field intensity (color: red to blue) at and around each of the OM arrays (near-field and far-field) for comparison with the results of coherent imaging. The nanoparticles in (j–l) are gray filled circles. Scale bars are 1.0 μm. See Supporting Videos 4 and 5 for a six-particle triangular OM array obtained with incoherent and coherent light, respectively.

We also recorded images of small 2D Ag nanoparticle OM arrays illuminated by spatially coherent light. Figure 2a–c shows aligned and averaged incoherent images for three different arrays. The associated averaged coherent dark-field illumination images are shown in Figure 2d–f. A real-space lattice fitting procedure was employed to detect ordered arrays and define the rotation and translation required for the averaging of each raw experimental image (see Supporting Information for details). The OM array in Figure 2a is a 6-particle triangular configuration as shown by the incoherent darkfield image. The positions of the corner particles are bright in the corresponding coherent image, while the positions of the three interior particles are dimer by comparison. Moving away from the array, bright fringes are visible in the coherent image of Figure 2d with maximum intensity located outward from the three central particles in the triangle. The array in Figure 2b is a different six-particle arrangement (termed a chevron) with a concave edge. Its coherent image in Figure 2e contains a smooth, bright fringe following the arc of positions of the outer particles, with the center particle appearing dark. There are exterior fringes projected outward from the bisectors of each of the 5 exterior edges of the array and a bright spot located at the 3 o’clock position. Figure 2c shows the incoherent image for the 7-particle hexagonal array obtained by adding a particle to the array in Figure 2b. The coherent image in Figure 2f is annular with a dark center that resembles a benzene π-orbital.^50^ There are fringes arranged parallel to each edge of the hexagon. The images in Figure 2a–c and d–f have 3-fold, 2-fold, and 6-fold rotational symmetry, respectively, which matches the symmetry of each particle array. Figure 2g–i shows simulated coherent backscattering images (λ = 800 nm; NA = 1.00) for each of the experimentally measured arrays in Figure 2a–c. The agreement between the measured and simulated images is very good.

Figure 2j–l shows the simulated electric field intensity |*E*|^2^ at and around each of the three arrays for comparison with the experimental and simulated coherent backscattering images. Comparison of the coherent images in Figure 2d–f (experimental) and g–i (simulated) with the electric field distributions in Figure 2j–l shows that they are clearly different inside the OM array but become more similar moving outward. Figure 2j exhibits two local intensity maxima outside each edge of the triangle that are in a similar location to the bright fringes in the experimental and simulated coherent images. The intensity maxima just outside of the array in Figure 2k,l are also coincident with fringes in the measured and simulated coherent images.

The electric field intensity distribution is related to the coherent images of OM arrays by far-field interference.^57^ For plane-wave illumination with incident electric field ***E***_0_ and wavevector *k*, the electric field intensity at a point (ρ, ϕ) in the transverse plane is given by^46^ (see Supporting Information for derivation)
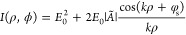
1where  is a complex constant related to the nanoparticle’s polarizability, and φ_s_ is a phase shift factor. Meanwhile, the field in the image plane scattered by a point dipole μ_*i*_ located at the origin (in the paraxial limit) is^46^
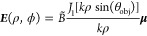
2where  is a complex constant, *J*_1_ is a Bessel function, and θ_obj_ is the collection angle of the microscope objective. Replacing the Bessel function by its asymptotic form and including the electric field reflected off the water–glass interface, ***E***_*r*_, the intensity is
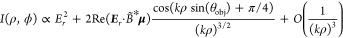
3

Comparing eq 3 to eq 1 (and ignoring the |μ|^2^ term) shows that for a perfect objective (sin(θ_obj_) = 1; NA = 1.33), the coherent images and the electric field intensity for a single particle have identical features up to a constant phase shift, although the image intensity modulation falls off faster, as ρ^3/2^. Eqs 1–3 apply to single particles. The difference between the coherent images and the near-field intensity in the interior of the array is due to the limited NA of our experimental coherent images.

### Multiparticle Scattering, Induced Polarization, and Coupling in OM Arrays

Figure 1 and Video 3 demonstrate that the separation between particles has a dramatic effect on the images of coherent light scattered by a pair of particles. Figure 2 demonstrates that the size and shape of the OM array do as well. However, the relative importance of interference and coupling in various characteristics of OM arrays needs to be established.

**Figure 3:**
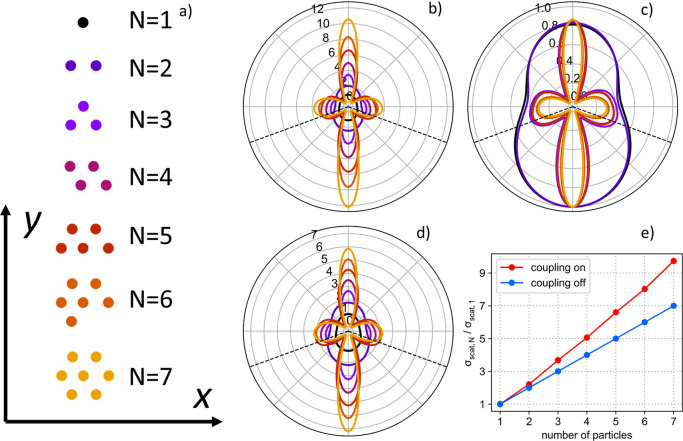
Effect of electrodynamic coupling as seen in projections of the far-field angular scattering (λ = 800 nm; 600 nm in water) onto the *yz* plane from NP arrays with 1–7 particles. (a) OM arrays (lattice spacing = 600 nm) and corresponding color coding for (b–d). The incident field in (b–d) propagates along the *z* direction (upward on the page). (b) Angular scattering normalized by the number of particles in the array. (c) Angular scattering normalized to unity. (d) Same as (b), but with interparticle electrodynamic interactions (i.e., coupling) disabled. Comparing (b) to (d) shows that coupling increases the strength of the far-field scattering. On the other hand, coupling does not significantly change the shape of the angular fields. (e) Total scattering normalized by single particle scattering with coupling enabled (red) and disabled (blue). In simulations where coupling is enabled, the scattering increases super extensively.

We performed GMMT calculations at a wavelength of 800 nm (600 nm in water) for ordered OM arrays with a lattice spacing of 600 nm with 1–7 particles to facilitate a quantitative comparison between the light scattered by OM arrays with different numbers of particles. The induced polarization of a particle in an OM structure can be calculated by taking the 2-norm of the electric dipole of the nanoparticle (see Supporting Information). From this, we can calculate the averaged induced polarization and the enhancement of the induced polarization, the expressions for which are in Supporting Information. The simulated OM arrays have the structures and orientations shown in Figure 3a. Projections of the scattered intensity onto the *y*–*z* plane are shown in Figure 3b,c when (c) normalized to 1 and (b) by the number of particles. Full 3D far-field scattering profiles for 1–7 particles are shown in Supporting Information.

A single particle scatters in all directions, although there is a greater scattering intensity in the forward and backward (±*z*; up/down) directions than in the sideways (*y*; left/right) directions. The pattern is reminiscent of a dipole emitter oriented perpendicular to the incident beam propagation direction. However, it is altered due to the higher-order (e.g., quadrupole) modes of the generalized Mie theory description of a single 150 nm diameter Ag NP and by the broken symmetry between forward and backward scattering. As more particles are added to the OM array, the scattering intensity develops a strong lobe structure with maxima in the forward (+*z*) and backward (−*z*) directions and smaller maxima in the sideways (±*y*) directions. The change going from 2 to 3 particles is particularly striking and notable because this is the first array where a particle is added offset from the *x* axis and also where multiparticle scattering and many-body induced polarizations occur.

Figure 3b,c shows that the directional scattering from an OM array is altered significantly compared to a single particle when electrodynamic interactions (i.e., coupling) are enabled. Figure 3d,e shows the effect of disabling coupling (i.e., induced polarization from particle–particle scattered fields) between the particles so that the polarization induced in each particle is only due to the incident field. The shape of the angular scattering profile is nearly identical, but the magnitude (Figure 3d) is 2-fold smaller than that when coupling is allowed (Figure 3b). Specifically, Figure 3e shows that the total scattering cross-section, σ_*N*_, (at a wavelength matching the lattice spacing) of an OM array with *N* particles is directly proportional to *N* (σ_*N*_ = *N*σ_1_) with coupling disabled, while σ_*N*_ grows superextensively (i.e., faster than *N*) with electrodynamic coupling enabled.

We also repeated our calculations of coherent images with coupling disabled to determine whether the images are affected. We find that the resulting images are nearly identical to the results shown in Figures 1 and 2 with coupling enabled (see Supporting Information). Essentially, only the total scattered intensity changes (increases) with coupling.

The results in Figures 1 through 3 demonstrate that the imaging and directionality of light scattered by OM arrays are primarily influenced by interference and that electrodynamic coupling changes the magnitude but not the spatial characteristics of the scattered coherent light. There are two (limiting) cases, where electrodynamic coupling between nanoparticles is particularly important: (i) when interparticle separations are small compared to the wavelength of light, retardation can be neglected, and the interaction between particles can be treated as quasi-static; i.e., as between the surface charges of the two particles in a pair or dimer;^37,38^ (ii) on the other hand, large field enhancements can occur in extended, regularly spaced arrays of particles at wavelengths near the array spacing due to constructive interference.^39,58^

### Spectral Dependence of Electrodynamic Coupling

**Figure 4:**
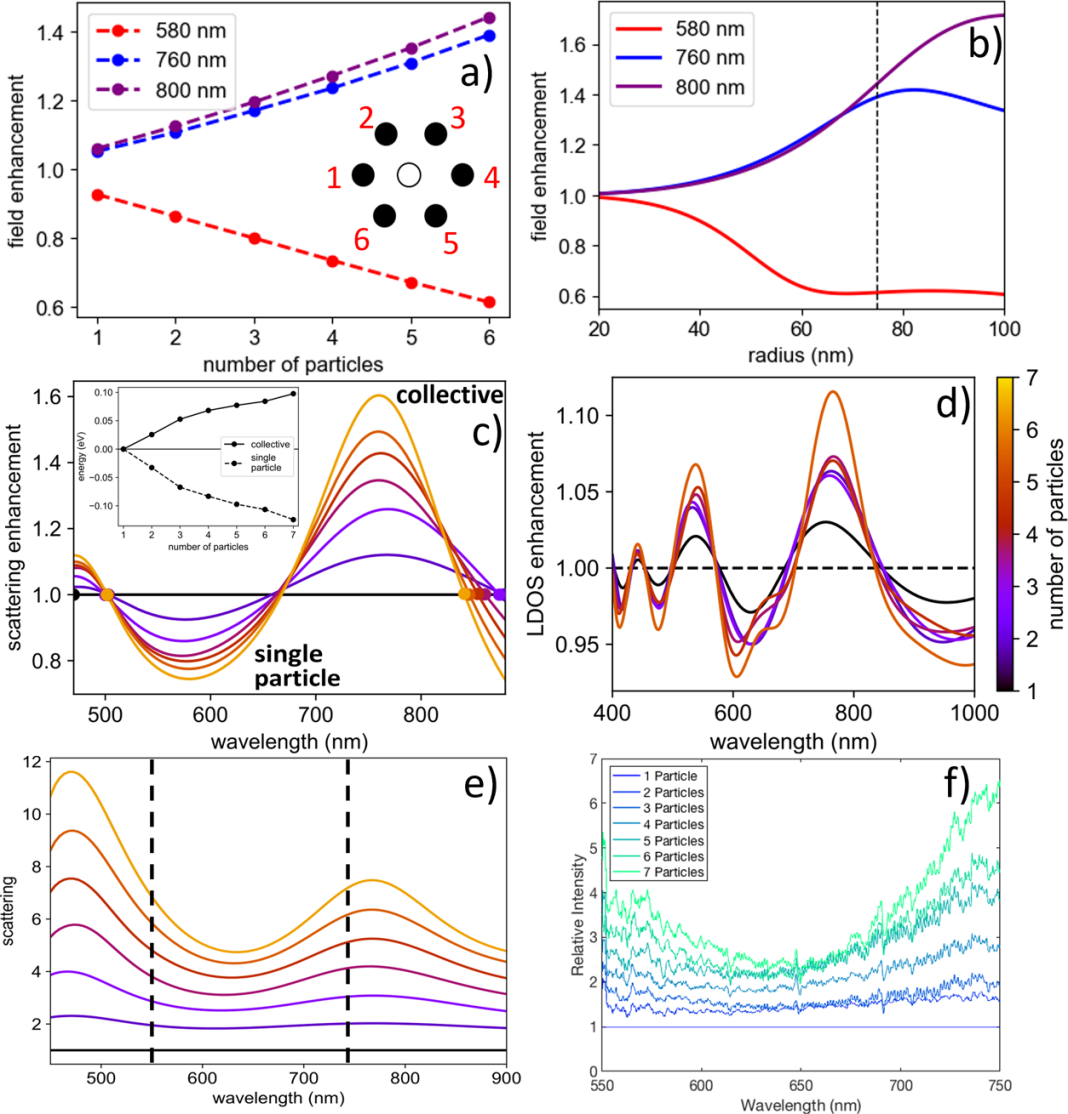
Electrodynamic coupling and emergence of a collective scattering mode in OM arrays. (a) Electric field enhancement at the vacant location of the center of a hexagonal OM array (open circle in (a)) for varying number of neighboring particles (filled circles in (a)) at incident vacuum wavelengths of 800 nm (violet), 760 nm (blue), and 580 nm (red). (b) Six-particle results from panel (a) repeated for varying particle sizes (radius) at wavelengths of 800 nm (violet), 760 nm (blue), and 580 nm (red). (c) Simulated scattering enhancement as a function of wavelength in OM arrays versus number of particles. The inset shows the integrals for the wavelength ranges corresponding to (i) single-particle Mie resonance and (ii) collective mode resonance resulting from electrodynamic coupling. (d) Local density of (electromagnetic) states enhancement in OM arrays for 1–7 particles. The results in (d) are on a per-particle basis. (e) Simulated wavelength-dependent total scattering of the NP arrays normalized by the particle number. Note that the spectral range of the experiment corresponds to that between the dashed vertical lines. (f) Experimental backscattering spectra of NP arrays normalized by a 1 NP spectrum measured with spatially coherent light.

We have shown that electrodynamic coupling, where the induced polarization is influenced by the fields scattered between particles, leads to increased scattering of coherent light at the trapping laser wavelength (800 nm; 600 nm in water) in OM arrays and now turn our attention to the origin of the coupling. We carried out GMMT calculations to study the effects of the number of particles, size of particles, and excitation wavelength on the coherent light scattered by OM arrays. Figure 4a shows the ratio of the total field to the incident field at the (empty) location of the center particle in a hexagonal six-particle OM array for vacuum wavelengths of 800 nm (violet), 760 nm (blue), and 580 nm (red). For λ = 800 nm and λ = 760 nm, the enhancement is small (≈7 percent) with a single particle nearby. However, every particle added to the array contributes to a growing enhancement so that the scattered field is approaching half the magnitude of the incident field for 6 nearby particles and the growth from 1 to 6 is nonlinear. Conversely, at λ = 580 nm, the total field at the location of the vacant site at the center of the OM array diminishes monotonically with increasing particle number.

Figure 4b shows the six-particle GMMT simulation of field enhancement at the vacant site as a function of particle radius ranging from 20 to 100 nm for the same three wavelengths as in Figure 4a. The dependence of the field enhancement on particle size is nonlinear at each wavelength. The magnitude of the field enhancement at λ = 800 nm increases monotonically with particle size, while the field enhancement reaches a peak near the 80 nm radius at λ = 760 nm before decreasing slightly. Conversely, the strength of the electric field decreases with increasing particle size for λ = 580 nm. These results follow from changes in the scattering cross sections due to the nanoparticle volume changing ∝ *r*^3^, in addition to the dipolar resonance red shifting with increasing nanoparticle size. Together, Figure 4a,b show that significant electrodynamic coupling occurs even in small arrays (2–7 particles) due to the combined scattering from several neighbors for particles larger than ≈50 nm in radius.

Figure 4c shows the scattering enhancement of spatially coherent broadband light (compared to *N*-fold multiplication of single-particle scattering) as a function of wavelength for OM arrays with 1–7 particles. Consistent with the results in Figure 3e, the scattering grows superextensively at wavelengths near the trapping laser wavelength. The dependence of this scattering enhancement on electrodynamic coupling suggests that it is collective in nature. Figure 4c also shows that the scattering near the single-particle Mie resonance decreases as the number of nanoparticle constituents in the OM array increases. The inset in Figure 4c shows the integral of the scattering enhancement for the collective and single-particle resonances. As the number of particles increases, the integral of the collective resonance enhancement steadily increases, while the integral of the single-particle resonance diminishes.

The local density of (electromagnetic) states (LDOS) at a certain location within or near an OM array controls the emission rate of a dipole emitter placed at that location.^59,60^ In the limit of large arrays of plasmonic particles, the LDOS enhancement (for specific in-plane wave vectors) occurs together with large field enhancements.^40^Figure 4d shows the LDOS enhancement in an OM array for 1–7 nanoparticles, which is consistent with the significant field enhancement shown in Figure 4a,b. The LDOS enhancement increases prominently near the trapping laser wavelength as more particles are added to the OM array.

Figure 4e shows simulated backscattered spectra for an OM array consisting of 1–7 particles normalized by the single particle spectrum. Peaks in scattering enhancement emerge near 500 and 800 nm as particles are added to the array. The experimentally measurable range of wavelengths is indicated by the black vertical dashed lines in Figure 4e. Figure 4f shows the experimentally measured backscattered spectra normalized by the single-particle scattering spectrum. The experimental and simulated spectra of the OM arrays are in good agreement, thus verifying the collective scattering resonance.

### Induced Polarization and Electromagnetic Field Scattering Enhancement in Large OM Arrays

**Figure 5:**
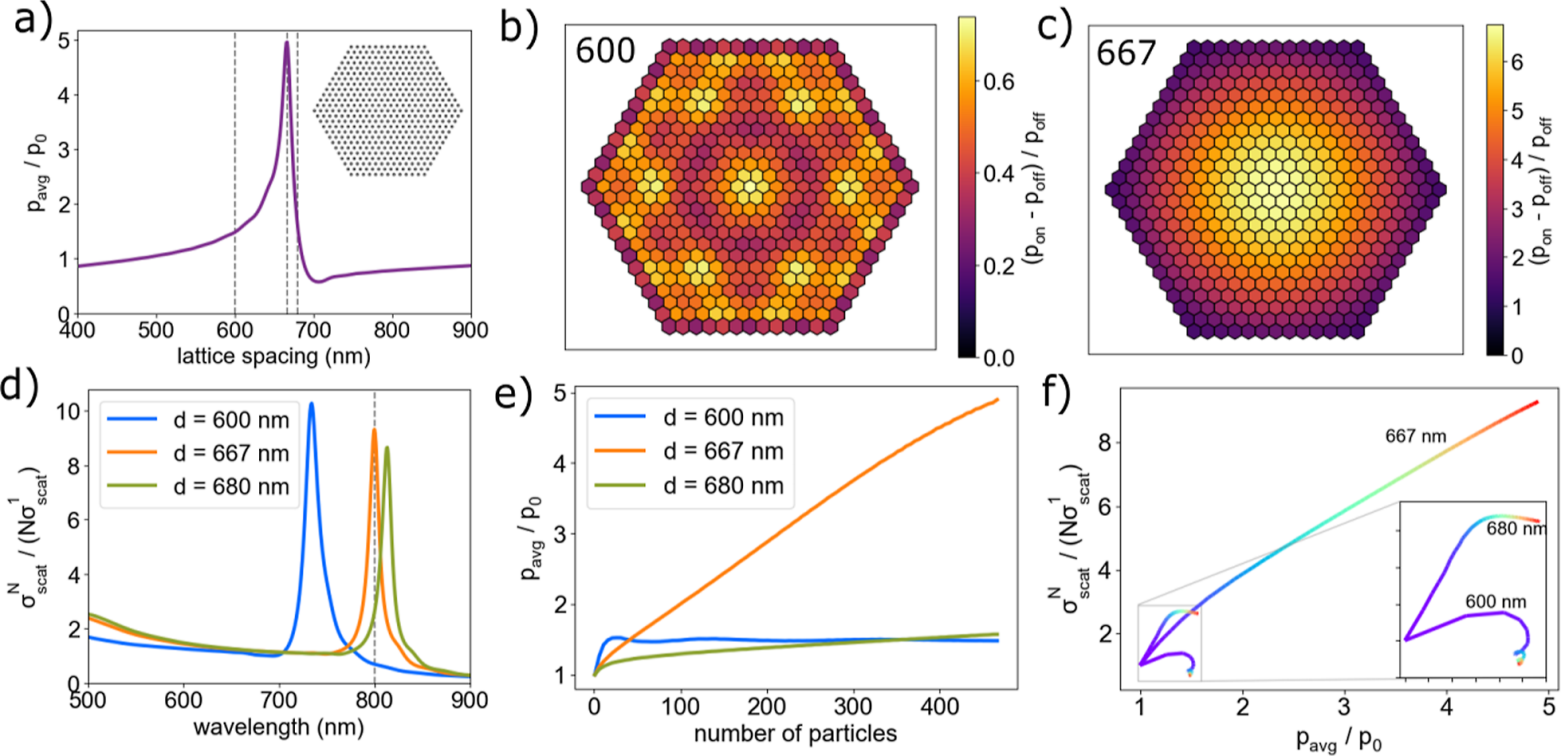
Induced-polarization and scattering enhancement in large hexagonal NP arrays. (a) Average induced-polarization enhancement in a large (469 particles) hexagonal NP array with variable interparticle spacing. The simulation is for λ = 800 nm (vacuum) and index of refraction of the medium, *n* = 1.33. The inset shows the arrangement of Ag nanoparticles. (b) Visualization of induced-polarization enhancement as a result of multiparticle scattering in a large hexagonal NP array with 600 nm spacing. (c) Visualization of induced-polarization enhancement in a large hexagonal NP array with 667 nm spacing. (d) Enhancement of the scattering cross section per particle for hexagonal arrays with lattice constants of *d* = 600, *d* = 667, and *d* = 680 nm. (e) Average induced-polarization enhancement of NP’s in a hexagonal array as it is built particle-by-particle for lattice constants of *d* = 600, *d* = 667, and *d* = 680 nm. (f) Enhancement of the scattering cross section per particle versus average polarization-enhancement of NP’s in a hexagonal array as it is built particle-by-particle indicated by the purple to red color scale for lattice constants of *d* = 600, *d* = 667, and *d* = 680 nm.

We extended our investigation to large hexagonal arrays of 150 nm Ag NP’s to elucidate the connection between the electrodynamic properties of small OM arrays and SLR’s in the infinite lattice limit. Figure 5a shows the average enhancement of the induced polarization (i.e., the ratio of the average induced polarization of the particles to the induced polarization of an isolated particle in the same incident field) in a hexagonal NP array with 469 particles and a lattice constant varying from 400 to 900 nm (in a simulated water environment with index of refraction *n* = 1.33 and a vacuum wavelength of λ = 800 nm). The enhancement of the induced polarization, and hence electrodynamic coupling as a result of multiparticle scattering, grows slowly for lattice spacings from 400 to 600 nm before rapidly increasing to a peak at 667 nm. It then declines rapidly to a value below 1 and then increases back toward 1 with increasing spacing. Figure 5b,c shows visualizations of the induced-polarization enhancement of the particles in the array for lattice spacings of 600 and 667 nm, respectively. At 600 nm, the induced-polarization enhancement has a 6-fold symmetric pattern and is small. At 667 nm (the spacing where the induced-polarization enhancement is maximized), the maximum enhancements are nearly radially symmetric, with the strongest (nearly 10-fold) enhancements at the center of the array.

We simulated scattering spectra for arrays with three separations marked with vertical dashed lines. Figure 5d shows the results obtained from GMMT simulations of 469-particle hexagonal arrays (in water) for wavelengths between 500 and 900 nm. The resonance peaks occur at 733, 799, and 812 nm for lattice spacings of 600 nm (blue), 667 nm (orange), and 680 nm (green), respectively. There is a nearly linear relationship between the resonance wavelength and lattice spacing over the range of OM structures studied.

We also conducted GMMT simulations as NP arrays were built particle-by-particle to investigate how the electrodynamic properties of the arrays scale with the number of nanoparticle elements, N. Figure 5e shows plots of induced-polarization enhancement versus the number of particles. When *d* = 600 nm (i.e., the laser wavelength is equal to the interparticle spacing), the induced-polarization enhancement increases rapidly for a small number of particles before leveling off and decreasing slightly. For *d* = 667 nm, the polarization enhancement increases more slowly for a small number of particles compared to *d* = 600 nm but continues to increase steadily, becoming 4-fold larger than the result for *d* = 600 nm with 469 particles. In contrast, the induced-polarization enhancement increases only slightly at *d* = 680 nm.

Figure 5f shows plots of scattering enhancement versus polarization enhancement for hexagonal arrays with a varying number of particles (indicated by the purple-to-red color scale) for a simulated incident wavelength of 800 nm and lattice spacings of *d* = 600, *d* = 667, and *d* = 680 nm. The scattering enhancement increases steadily and monotonically with the polarization enhancement for *d* = 667 nm. However, scattering enhancement increases up to a certain number of particles before decreasing for *d* = 600 and *d* = 680 nm. These contrasting behaviors indicate that an increase in induced polarization does not necessarily result in increased total scattering. The phase of the induced polarizations of the particles in the array (see Supporting Information) shows that the collective excitation in the 469-particle array: (i) lags behind the phase of the incident light for *d* = 600 nm; (ii) is close to the phase of the incident light for *d* = 667 nm; and (iii) is advanced compared to the phase of the incident light for *d* = 680 nm.

## Discussion and Conclusions

We have shown that electrodynamic coupling and associated induced polarizations in small (*N* = 1–7) metal nanoparticle-based OM arrays have distinct effects on the scattering of coherent light by OM arrays versus single scattering and interference in the absence of such electrodynamic interactions. Figure 1 shows that imaging the backscattering of the spatially coherent trapping laser from an OM array gives dramatically different results versus imaging the particles illuminated by an incoherent source. Furthermore, the coherent images of the OM arrays have some features in common with the near-field electromagnetic field intensity because both are controlled by similar phase-dependent relationships according to eqs 1 and 3. Figure 3 shows that multiple scattering and electrodynamic coupling do not significantly affect the (qualitative) spatial characteristics of coherent light scattered by OM arrays, i.e., how the images look.

However, Figure 4 demonstrates that coupling leads to an enhancement of total scattering at the trapping laser wavelength, but total scattering is not enhanced at all wavelengths. Figure 5 shows how the electrodynamic properties of OM arrays evolve as the arrays grow. For large hexagonal arrays, the collective scattering resonance wavelength (in a water medium with *n* = 1.33) is significantly shifted compared to the lattice spacing. However, our results show that maximization of scattering still occurs concurrently with large induced-polarization enhancements due to constructive interference of the light scattered by neighboring particles.

Figure 4a shows that each particle added to the OM array increases the electric field strength at the vacant central site of a hexagonal array for trapping laser wavelengths (λ/1.33) near the 600 nm (fixed) particle spacing. For the geometry and symmetry shown in Figure 4a, the light scattered from each particle has the same phase at the central location marked in that figure because that location is equidistant from all of the particles. The relative phase between the incident and scattered light, however, depends on the lattice spacing in comparison to the wavelength of the excitation. For the trapping laser, the laser wavelength (accounting for the index of refraction of the medium) and lattice spacing are nearly equal, and the scattered light interferes constructively with the incident light. At λ = 580 nm, the interference is destructive, and the field at the location of the vacant central site is diminished.

The total strength of the coupling also depends on the size and polarizability of the particles. Figure 4b shows that the scattering cross sections of the 150 nm diameter Ag nanoparticles used in our experiments and most calculations shown are large enough to result in significant field enhancement in OM arrays. However, Ag nanoparticles with diameters under 100 nm create almost none. Therefore, the geometry, interparticle separations, and choice of particles in OM arrays contribute to the significant electrodynamic coupling that we report here.

There is an important relationship between interference and coupling that can be understood within the point dipole approximation.^4^ Consider a two-dimensional array of particles with isotropic polarizability α arranged in the transverse plane of an electromagnetic plane wave with wavelength λ_0_. The induced polarization, **p**_*i*_, of particle i is proportional to the total electric field at the location of particle i,  with
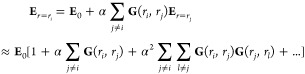
4where **E**_0_ is the incident electric field, and **G**(*r*_*i*_, *r*_*j*_) is the Green’s Function tensor that propagates the scattered field at position *r*_*j*_, resulting from a dipole at position *r*_*i*_(4,61)
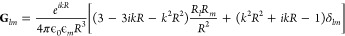
5where *l* and *m* are polarization directions of the field, *R* = |*r*_*i*_ – *r*_*j*_| is the distance between the two particles, and *k* is the wave-vector of the incident light. At single-wavelength distances *kR* = 2π the far-field terms in the propagator with  dependence give the largest contribution, although all terms are significant for OM systems. Due to the *e*^*ikR*^ phase factor in eq 5, the induced polarization of a particle in an OM array will have the largest contribution from light scattered by neighboring particles when all of the scattered contributions are in-phase, i.e., when they are interfering constructively.

Eq 4 is an approximate solution for the scattered field at the location of a particle in an OM array expanded in orders of scattering. Each scattering order is weaker by a factor of α*G*. Based on the results in Figure 4a, we estimate that the terms in α*G* are approximately 0.05 in magnitude. As an OM array grows, an increasing number of terms contribute to higher-order (and multiparticle) scattering. In the limit of large lattices, as demonstrated in Figure 5, higher-order and multiparticle scattering (and hence many-body interactions) and what we term electrodynamic coupling make the dominant contribution to the induced polarization of each particle, hence multiple-scattering and many-body effects cannot be ignored.

Both interference and electrodynamic coupling play important roles in understanding light scattered by OM arrays, analogous to SLR’s. Interference dramatically alters the spatial profile and directionality of the light scattered by the OM arrays. Furthermore, while the magnitude of the field scattered by a single particle is small compared to the incident field, the combined contributions from several nearby particles interfering constructively lead to significant field enhancement and coupling. This is especially true for large hexagonal arrays where the induced polarization becomes >5× larger than that of an isolated particle under the same illumination. These field enhancements and coupling could also be exploited for applications in nonlinear optics, where the phenomena have an *E*^2*n*^ dependence, with n indicating the order of nonlinearity.^62,63^

## Supporting Information Available

The coherent darkfield microscopy imaging of the optical matter system consisting of two 150 nm diameter Ag nanoparticles optically trapped in the water solution (Video 1) (AVI)
The incoherent darkfield microscopy imaging of two 150 nm diameter Ag nanoparticles optically trapped in the water solution, rotationally aligned and averaged for different interparticle distances (Video 2) (MP4)
The coherent darkfield microscopy imaging of two 150 nm diameter Ag nanoparticles optically trapped in the water solution, rotationally aligned and averaged for different interparticle distances (Video 3) (MP4)
The rotationally aligned and averaged incoherent darkfield microscopy imaging of the 6-particle triangle optical matter structure that consists of 150 nm diameter Ag nanoparticles (Video 4) (AVI)
The rotationally aligned and averaged coherent darkfield microscopy imaging of the 6-particle triangle optical matter structure that consists of 150 nm diameter Ag nanoparticles (Video 5) (AVI)
Details of the image averaging procedure used to enhance the signal-to-noise ratio of the experimental images; raw dark field and coherent images for several OM array configurations in experiment; coherent images and near-field intensity of a 7-particle OM array obtained by GMMT simulation with coupling disabled; derivation of expressions for the electric field intensity in the near-field and the image plane; far-field angular scattering profiles of OM arrays with number particles ranging from 1 to 7; experimental details of the collection of backscattered spectra; detailed evaluation of OM-induced polarization; and demonstration of the phase of the induced polarization of the OM array (PDF)

## Supplementary Material

jp3c08459_si_001.avi

jp3c08459_si_003.mp4

jp3c08459_si_004.avi

jp3c08459_si_005.avi

## References

[ref1] Tatarkova S. A.; Carruthers A. E.; Dholakia K. One-Dimensional Optically Bound Arrays of Microscopic Particles. Phys. Rev. Lett. 2002, 89, 283901 10.1103/PhysRevLett.89.283901. 12513147

[ref2] Burns M. M.; Fournier J. M.; Golovchenko J. A. Optical Binding. Phys. Rev. Lett. 1989, 63, 1233–1236. 10.1103/PhysRevLett.63.1233. 10040510

[ref3] Burns M. M.; Fournier J.-M.; Golovchenko J. A. Optical Matter: Crystallization and Binding in Intense Optical Fields. Science 1990, 249, 749–754. 10.1126/science.249.4970.749. 17756787

[ref4] Dholakia K.; Zemánek P. Colloquium: Gripped by Light: Optical Binding. Rev. Mod. Phys. 2010, 82, 1767–1791. 10.1103/RevModPhys.82.1767.

[ref5] Forbes K. A.; Bradshaw D. S.; Andrews D. L. Optical Binding of Nanoparticles. Nanophotonics 2020, 9, 1–17. 10.1515/nanoph-2019-0361.

[ref6] Yan Z.; Shah R. A.; Chado G.; Gray S. K.; Pelton M.; Scherer N. F. Guiding Spatial Arrangements of Silver Nanoparticles by Optical Binding Interactions in Shaped Light Fields. ACS Nano 2013, 7, 1790–1802. 10.1021/nn3059407. 23363451

[ref7] Demergis V.; Florin E.-L. Ultrastrong Optical Binding of Metallic Nanoparticles. Nano Lett. 2012, 12, 5756–5760. 10.1021/nl303035p. 23035835

[ref8] Bradshaw D. S.; Andrews D. L. Optically induced forces and torques: Interactions between nanoparticles in a laser beam. Phys. Rev. A 2005, 72, 033816 10.1103/PhysRevA.72.033816.

[ref9] Sukhov S.; Dogariu A. Non-Conservative Optical Forces. Rep. Prog. Phys. 2017, 80, 112001 10.1088/1361-6633/aa834e. 28762956

[ref10] Sukhov S.; Shalin A.; Haefner D.; Dogariu A. Actio et Reactio in Optical Binding. Opt. Express 2015, 23, 247–252. 10.1364/OE.23.000247. 25835671

[ref11] Yifat Y.; Coursault D.; Peterson C. W.; Parker J.; Bao Y.; Gray S. K.; Rice S. A.; Scherer N. F. Reactive Optical Matter: Light-Induced Motility in Electrodynamically Asymmetric Nano-Scale Scatterers. Light: Sci. Appl. 2018, 7, 105 10.1038/s41377-018-0105-y. 30564311 PMC6289991

[ref12] Hakobyan D.; Brasselet E. Left-Handed Optical Radiation Torque. Nat. Photonics 2014, 8, 610–614. 10.1038/nphoton.2014.142.

[ref13] Chen J.; Ng J.; Ding K.; Fung K. H.; Lin Z.; Chan C. T. Negative Optical Torque. Sci. Rep. 2014, 4, 6386 10.1038/srep06386. 25226863 PMC4165981

[ref14] Sule N.; Yifat Y.; Gray S. K.; Scherer N. F. Rotation and Negative Torque in Electrodynamically Bound Nanoparticle Dimers. Nano Lett. 2017, 17, 6548–6556. 10.1021/acs.nanolett.7b02196. 28961013

[ref15] Han F.; Parker J. A.; Yifat Y.; Peterson C.; Gray S. K.; Scherer N. F.; Yan Z. Crossover From Positive to Negative Optical Torque in Mesoscale Optical Matter. Nat. Commun. 2018, 9, 4897 10.1038/s41467-018-07376-7. 30459430 PMC6244235

[ref16] Parker J.; Peterson C. W.; Yifat Y.; Rice S. A.; Yan Z.; Gray S. K.; Scherer N. F. Optical Matter Machines: Angular Momentum Conversion by Collective Modes in Optically Bound Nanoparticle Arrays. Optica 2020, 7, 1341–1348. 10.1364/OPTICA.396147.

[ref17] Nan F.; Li X.; Zhang S.; Ng J.; Yan Z. Creating stable trapping force and switchable optical torque with tunable phase of light. Sci. Adv. 2022, 8, eadd6664 10.1126/sciadv.add6664. 36399578 PMC9674277

[ref18] Han F.; Yan Z. Phase Transition and Self-Stabilization of Light-Mediated Metal Nanoparticle Assemblies. ACS Nano 2020, 14, 6616–6625. 10.1021/acsnano.9b08015. 32422042

[ref19] Albaladejo S.; Sáenz J. J.; Marqués M. I. Plasmonic Nanoparticle Chain in a Light Field: A Resonant Optical Sail. Nano Lett. 2011, 11, 4597–4600. 10.1021/nl201996t. 21942220

[ref20] Roichman Y.; Grier D. G. Three-Dimensional Holographic Ring Traps. Proc. SPIE 2007, 6483, 64830F 10.1117/12.701034.

[ref21] Yan Z.; Sajjan M.; Scherer N. F. Fabrication of a Material Assembly of Silver Nanoparticles Using the Phase Gradients of Optical Tweezers. Phys. Rev. Lett. 2015, 114, 143901 10.1103/PhysRevLett.114.143901. 25910124

[ref22] Damková J.; Chvátal L.; Ježek J.; Oulehla J.; Brzobohatý O.; Zemánek P. Enhancement of the ‘Tractor-Beam’ Pulling Force on an Optically Bound Structure. Light: Sci. Appl. 2017, 7, 17135 10.1038/lsa.2017.135. PMC6107043 30839610

[ref23] Peterson C. W.; Parker J.; Rice S. A.; Scherer N. F. Controlling the Dynamics and Optical Binding of Nanoparticle Homodimers with Transverse Phase Gradients. Nano Lett. 2019, 19, 897–903. 10.1021/acs.nanolett.8b04134. 30624071

[ref24] Nan F.; Yan Z. Synergy of Intensity, Phase, and Polarization Enables Versatile Optical Nanomanipulation. Nano Lett. 2020, 20, 2778–2783. 10.1021/acs.nanolett.0c00443. 32134670

[ref25] Rodrigo J. A.; Angulo M.; Alieva T. All-Optical Motion Control of Metal Nanoparticles Powered by Propulsion Forces Tailored in 3D Trajectories. Photonics Res. 2021, 9, 1–12. 10.1364/PRJ.408680.

[ref26] Yang Y.; Ren Y.; Chen M.; Arita Y.; Rosales-Guzmán C. Optical Trapping With Structured Light: A Review. Adv. Photonics 2021, 3, 034001 10.1117/1.AP.3.3.034001.

[ref27] Brzobohaty O.; Chvatal L.; Jonas A.; Siler M.; Kanka J.; Jezek J.; Zemánek P. Tunable Soft-Matter Optofluidic Waveguides Assembled by Light. ACS Photonics 2019, 6, 403–410. 10.1021/acsphotonics.8b01331.

[ref28] Nan F.; Yan Z. Tuning Nanoparticle Electrodynamics by an Optical-Matter-Based Laser Beam Shaper. Nano Lett. 2019, 19, 3353–3358. 10.1021/acs.nanolett.9b01090. 31013096

[ref29] Brzobohatý O.; Chvátal L.; Zemánek P. Optomechanical properties of optically self-arranged colloidal waveguides. Opt. Lett. 2019, 44, 707–710. 10.1364/OL.44.000707. 30702716

[ref30] Han X.; Luo H.; Xiao G.; Jones P. H. Optically bound colloidal lattices in evanescent optical fields. Opt. Lett. 2016, 41, 4935–4938. 10.1364/OL.41.004935. 27805654

[ref31] Kudo T.; Yang S.-J.; Masuhara H. A Single Large Assembly with Dynamically Fluctuating Swarms of Gold Nanoparticles Formed by Trapping Laser. Nano Lett. 2018, 18, 5846–5853. 10.1021/acs.nanolett.8b02519. 30071730

[ref32] Tsipotan A. S.; Gerasimova M. A.; Slabko V. V.; Aleksandrovsky A. S. Laser-induced wavelength-controlled self-assembly of colloidal quasi-resonant quantum dots. Opt. Express 2016, 24, 11145–11150. 10.1364/OE.24.011145. 27409936

[ref33] Parker J.; Nagasamudram S.; Peterson C.; Soleimanikahnoj S.; Rice S. A.; Scherer N. F. Symmetry Breaking Induced Many-Body Electrodynamic Forces in Optical Matter Systems. In preparation 2024. 10.1038/s41467-025-61616-1 PMC12238463 40628715

[ref34] Lamprecht B.; Schider G.; Lechner R. T.; Ditlbacher H.; Krenn J. R.; Leitner A.; Aussenegg F. R. Metal Nanoparticle Gratings: Influence of Dipolar Particle Interaction on the Plasmon Resonance. Phys. Rev. Lett. 2000, 84, 4721–4724. 10.1103/PhysRevLett.84.4721. 10990780

[ref35] Kravets V. V.; Yeshchenko O. A.; Gozhenko V. V.; Ocola L. E.; Smith D. A.; Vedral J. V.; Pinchuk A. O. Electrodynamic Coupling in Regular Arrays of Gold Nanocylinders. J. Phys. D: Appl. Phys. 2012, 45, 045102 10.1088/0022-3727/45/4/045102.

[ref36] Pinchuk A. O.; Schatz G. C. Nanoparticle Optical Properties: Far-and Near-Field Electrodynamic Coupling in a Chain of Silver Spherical Nanoparticles. Mater. Sci. Eng., B 2008, 149, 251–258. 10.1016/j.mseb.2007.09.078.

[ref37] Nordlander P.; Oubre C.; Prodan E.; Li K.; Stockman M. Plasmon Hybridization in Nanoparticle Dimers. Nano Lett. 2004, 4, 899–903. 10.1021/nl049681c.

[ref38] Jain P. K.; El-Sayed M. A. Plasmonic Coupling in Noble Metal Nanostructures. Chem. Phys. Lett. 2010, 487, 153–164. 10.1016/j.cplett.2010.01.062.

[ref39] García de Abajo F. J. Colloquium: Light Scattering by Particle and Hole Arrays. Rev. Mod. Phys. 2007, 79, 1267–1290. 10.1103/revmodphys.79.1267.

[ref40] Wang W.; Ramezani M.; Väkeväinen A. I.; Törmä P.; Rivas J. G.; Odom T. W. The Rich Photonic World of Plasmonic Nanoparticle Arrays. Mater. Today 2018, 21, 303–314. 10.1016/j.mattod.2017.09.002.

[ref41] Kravets V. G.; Kabashin A. V.; Barnes W. L.; Grigorenko A. N. Plasmonic Surface Lattice Resonances: A Review of Properties and Applications. Chem. Rev. 2018, 118, 5912–5951. 10.1021/acs.chemrev.8b00243. 29863344 PMC6026846

[ref42] Cherqui C.; Bourgeois M. R.; Wang D.; Schatz G. C. Plasmonic Surface Lattice Resonances: Theory and Computation. Acc. Chem. Res. 2019, 52, 2548–2558. 10.1021/acs.accounts.9b00312. 31465203

[ref43] Rodriguez S. R. K.; Schaafsma M. C.; Berrier A.; Gómez Rivas J. Collective Resonances in Plasmonic Crystals: Size Matters. Phys. B 2012, 407, 4081–4085. 10.1016/j.physb.2012.03.053.

[ref44] https://github.com/johnaparker/miepweby, 2023.

[ref45] Bohren C. F.; Huffman D. R. Absorption and Scattering of Light by Small Particles; John Wiley & Sons, 2008.

[ref46] Novotny L.; Hecht B. Principles of Nano-Optics; Cambridge University Press, 2012.

[ref47] Zou S.; Janel N.; Schatz G. C. Silver Nanoparticle Array Structures That Produce Remarkably Narrow Plasmon Lineshapes. J. Chem. Phys. 2004, 120, 10871–10875. 10.1063/1.1760740. 15268116

[ref48] Sherry L. J.; Chang S.-H.; Schatz G. C.; Van Duyne R. P.; Wiley B. J.; Xia Y. Localized Surface Plasmon Resonance Spectroscopy of Single Silver Nanocubes. Nano Lett. 2005, 5, 2034–2038. 10.1021/nl0515753. 16218733

[ref49] Yan Z.; Gray S. K.; Scherer N. F. Potential Energy Surfaces and Reaction Pathways for Light-Mediated Self-Organization of Metal Nanoparticle Clusters. Nat. Commun. 2014, 5, 3751 10.1038/ncomms4751. 24786197

[ref50] Moore J. T.; Stanitski C.; Jurs P. C. Principles of Chemistry: The Molecular Science; Cengage Learning, 2009.

[ref51] Xu Y.-L. Electromagnetic Scattering by an Aggregate of Spheres. Appl. Opt. 1995, 34, 4573–4588. 10.1364/AO.34.004573. 21052290

[ref52] Mackowski D. W.; Mishchenko M. I. Calculation of the T Matrix and the Scattering Matrix for Ensembles of Spheres. J. Opt. Soc. Am. A 1996, 13, 2266–2278. 10.1364/JOSAA.13.002266.

[ref53] Parker J. A. Collective Electrodynamic Excitations and Non-conservative Dynamics in Optical Matter and Meta-Atom Systems. Ph.D. thesis, University of Chicago, 2020.

[ref54] Mie G. Beiträge zur Optik trüber Medien, speziell kolloidaler Metallösungen. Ann. Phys. 1908, 330, 377–445. 10.1002/andp.19083300302.

[ref55] Stein S. Addition Theorems for Spherical Wave Functions. Q. Appl. Math. 1961, 19, 15–24. 10.1090/qam/120407.

[ref56] Araneda G.; Walser S.; Colombe Y.; Higginbottom D. B.; Volz J.; Blatt R.; Rauschenbeutel A. Wavelength-Scale Errors in Optical Localization Due to Spin–Orbit Coupling of Light. Nat. Phys. 2019, 15, 17–21. 10.1038/s41567-018-0301-y. 30854021 PMC6398575

[ref57] Wild B.; Cao L.; Sun Y.; Khanal B. P.; Zubarev E. R.; Gray S. K.; Scherer N. F.; Pelton M. Propagation Lengths and Group Velocities of Plasmons in Chemically Synthesized Gold and Silver Nanowires. ACS Nano 2012, 6, 472–482. 10.1021/nn203802e. 22185403

[ref58] Hicks E. M.; Zou S.; Schatz G. C.; Spears K. G.; Van Duyne R. P.; Gunnarsson L.; Rindzevicius T.; Kasemo B.; Käll M. Controlling Plasmon Line Shapes Through Diffractive Coupling in Linear Arrays of Cylindrical Nanoparticles Fabricated by Electron Beam Lithography. Nano Lett. 2005, 5, 1065–1070. 10.1021/nl0505492. 15943444

[ref59] Purcell E. M.; Torrey H. C.; Pound R. V. Resonance Absorption by Nuclear Magnetic Moments in a Solid. Phys. Rev. 1946, 69, 37–38. 10.1103/PhysRev.69.37.

[ref60] Pelton M. Modified Spontaneous Emission in Nanophotonic Structures. Nat. Photonics 2015, 9, 427–435. 10.1038/nphoton.2015.103.

[ref61] Jackson J. D. Classical Electrodynamics, 3rd ed.; John Wiley & Sons, Inc.: Hoboken, NJ, 1999.

[ref62] Shen Y.-R. The Principles of Nonlinear Optics; John Wiley & Sons, Inc.: Hoboken, NJ, 2003.

[ref63] Jin R.; Jureller J. E.; Scherer N. F. Precise Localization and Correlation of Single Nanoparticle Optical Responses and Morphology. Appl. Phys. Lett. 2006, 88, 263111 10.1063/1.2213518.

