# Supplementary material for: Electrodynamic Interference and Induced Polarization in Nanoparticle-Based Optical Matter Arrays
Source: J Phys Chem C Nanomater Interfaces. 2024 Apr 26;128(18):7560–71. doi: 10.1021/acs.jpcc.3c08459 (PMC11089571; doi:10.1021/acs.jpcc.3c08459)
Supplement: Supplementary file 6 — jp3c08459_si_006.pdf [file jp3c08459_si_006.pdf]

Supplementary Information for Electrodynamic Interference and Induced-Polarization in Nanoparticle-based Optical Matter Arrays

Curtis Peterson[1,2], John Parker[2,3], Emmanuel Valenton[1,2], Yuval Yifat[†2], Shiqi Chen[1,2], Stuart A. Rice[1,2], and Norbert F. Scherer[*,1,2]

[1] *Department of Chemistry, The University of Chicago, Chicago, IL 60637, USA*

[2] *James Franck Institute, The University of Chicago, Chicago, IL 60637, USA*

[3] *Department of Physics, The University of Chicago, Chicago, IL 60637, USA*

[*] E-mail : nfschere@uchicago.edu

1 Image averaging procedure

The signal to noise ratio (SNR) of the experimental images of Ag nanoparticle dimers shown in the manuscript was enhanced through averaging. Averaging images presents a challenge due to translational and rotational thermal motion of the particle pair in the optical trap. This was addressed by translating each image so that the center-point (*i.e.* center of mass) between the two particles is in the center of the image (*i.e.*, centered on the coordinate system) and then rotating the translated image to a common orientation. The positions of the particles in the darkfield image were found using single-particle tracking (ImageJ plug-in Mosaic, which uses an implementation of the Crocker-Grier algorithm [1,2,3]). Single-particle tracking was generally not possible in "coherent" images (*i.e.*, those measured using coherent laser light) due to overlapping features and lack of precise relationship to the nanoparticles (see Figure 1d,e in main text). Therefore, the positions of the particles in the coherent images were calculated by adding offset values (in x, y) to the particle positions in the darkfield images that were determined by comparing the position of the center of mass of the dimer in the darkfield and coherent images for all frames where the two particles were sufficiently separated such that there were no overlapping features.

[†] Current adddress: Innoviz Technologies Ltd 2 Amal Street, Rosh Ha'Ayin, Israel

The built-in MATLAB function **imrotate** was used to rotate each translated image, and the rotated images were cropped or padded so that they are all the same size. The image averaging was performed once each frame (i.e., image) in the experimental video is translated, rotated, and cropped. The inter-particle separation for each frame was calculated from the particle positions tracked in the raw dark-field images. All translated, rotated, and cropped images where interparticle separation, r, was within an interval $[r_{bin} - \delta, r_{bin} + \delta]$ were averaged together. In particular, we set $r_{bin} = 600$ nm and $r_{bin} = 900$ nm with $\delta = 100$ nm to obtain the coherent and incoherent light scattering images shown in Figure 1. Note that this value of δ and the associated averaging of images will decrease the apparent spatial resolution of the imaging system as judged by comparison with the simulated images and warrants the reduced value of the NA used in obtaining comparable simulation images.

To illustrate the procedure, Figure S1 shows a raw experimental coherent image. In Figure S1a, a single frame taken from a long video, the separation between particles was measured to be $852nn$. Figure S1b shows the same image, but translated, rotated, and cropped so that the center-point of the dimer lies in the center of the image and the dimer lies on the horizontal axis. Figure S1c shows the average of 157 images where r was in the interval $[800nm, 1000nm]$.

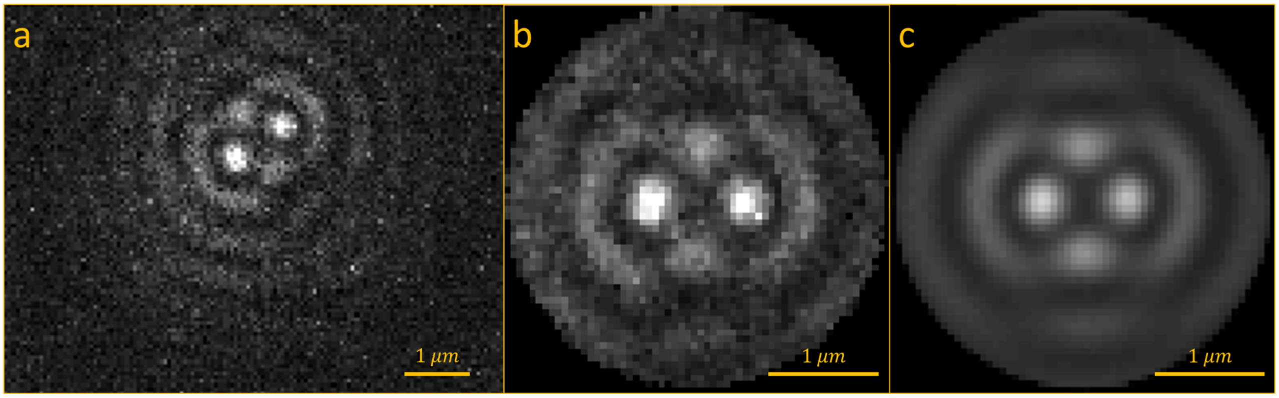

Figure S1: Steps in averaging procedure for a dimer **(a)** Raw coherent image of a dimer **(b)** Translated, cropped, and rotated coherent image of a dimer **(c)** Average of several translated, cropped, and rotated coherent images of a dimer with inter-particle separations between $800nm$ and $1000nm$.

Image-averaging for the small 2D optical matter (OM) arrays shown in the main text was also performed using a real-space lattice fitting procedure described in Chen *et al.*[4]. The first step was to find the best-fit hexagonal lattice to the particle positions determined in each frame of dark-field microscopy video obtained with incoherent light. Figure S2 shows an example of a best-fit lattice for a 7-particle hexagonal OM array configuration. Once the best-fit lattice was found, each detected

particle was assigned to a particular lattice site. A neighbor vector, $\boldsymbol{v}$, defined as

$$\boldsymbol{v} = \begin{bmatrix} v_1 \\ \vdots \\ v_6 \end{bmatrix} \tag{S1}$$

where v_n is the number of lattice sites with n neighbors was used to differentiate between different configurations of OM arrays. For example, for the hexagonal 7-particle configuration shown in Figure S2 $\boldsymbol{v} = [0, 0, 6, 0, 0, 1]$. The videos were then sorted by the OM array configuration associated with the lattice site neighbor vector, and the images were centered and rotated (i.e., aligned). The center-point of the images was chosen as the average position of the occupied lattice sites, while the rotation angle shown in Figure S2 as θ_{rot} was chosen as the angle between the x-axis and one of the lattice vectors of the best-fit lattice.

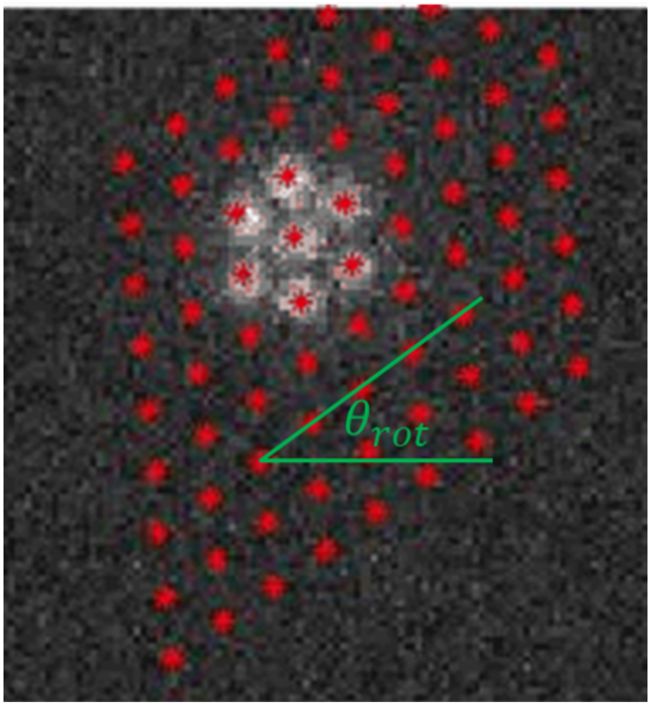

Figure S2: Best-fit lattice to a hexagonal 7-particle OM array and illustration of θ_{rot}, *i.e.*, the angle between the lattice vector and the x-axis. The lattice is denoted by the array of red spots. Note the overlap with the 7 nanoparticles (white spots) measured by dark-field microscopy with incoherent light.

2 Raw darkfield and coherent images for several OM array configurations

Figure S3 shows raw (single frame, not averaged) darkfield and coherent images for several different types of OM arrays. Figures S3a-d show darkfield images of an OM array building up from 6 to 9 particles by adding a single 150 nm diameter Ag nanoparticle at a time. Figures S3e-h show the corresponding coherent images. As the OM array is built up, changes in both the central bright region and exterior fringes are observed. Figures S3i-l show an alternative assembly pathway for an OM array built up from 6 to 9 particles, and Figures S3m-p show the corresponding coherent images. Similar to the first assembly pathway, there are significant changes to the coherent images as more particles are added. However, the coherent images for the two assembly pathways are distinct from one another despite differing by only a single particle in most cases. The most prominent characteristic of the coherent images is that they share the same symmetry as the underlying OM array.

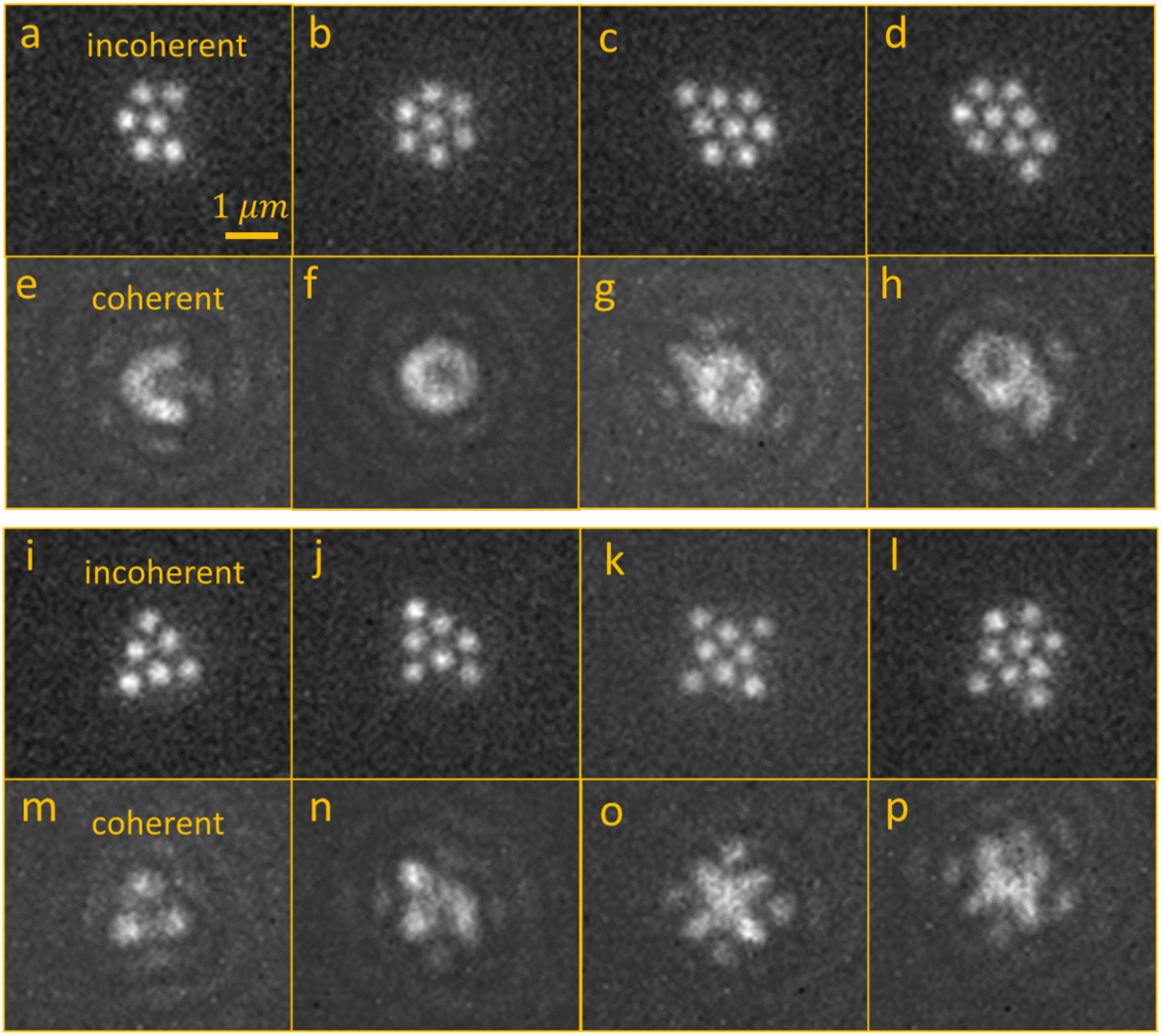

Figure S3: Darkfield incoherent and coherent images of OM arrays with 6-9 particles. These images are single frames in videos and are not averaged. **(a-d)** Darkfield images of an OM array built up from 6 to 9 particles by adding a single particle at a time. **(e-h)** Coherent images corresponding to the configurations shown in panels **(a-d)**. **(i-l)** Darkfield images of an OM array built up from 6 to 9 particles along an alternative assembly pathway. **(m-p)** Coherent images corresponding to the configurations shown in panels **(i-l)**.

3 Simulated coherent images and near-field intensity with coupling disabled

Figure S4 shows a comparison of the coherent images and the near-field electric field intensity for OM arrays with electrodynamic coupling disabled (*i.e.* the induced-polarization of each particle has no contribution from the light scattered by other nearby particles) and enabled. Figures S4a-b show simulated coherent images for 6 and 7 NP OM arrays, respectively. The spatial profiles of the coherent images are nearly identical to those obtained from full generalized multiparticle Mie theory (GMMT) simulations, although there are small differences in relative intensity (e.g., the fringes outside the arrays are relatively diminished). Figures S4c-d show the near-field electric field intensity throughout the same 6 and 7 NP OM arrays. The spatial profile is once again similar to the results obtained from full GMMT simulations (*i.e.*, with electrodynamic coupling enabled). Figures S4e-h (reproduced from Figure 2 in the main text) show the same images as Figures S4a-d but with coupling enabled for comparison.

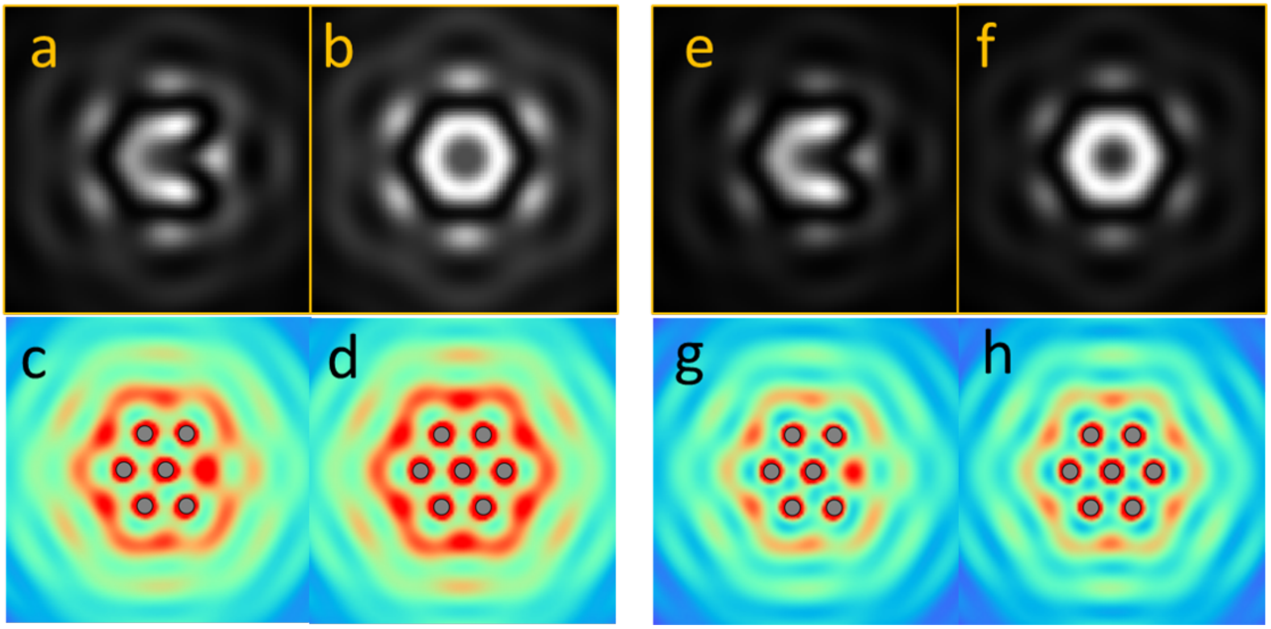

Figure S4: **(a-b)** Simulated coherent images for (a) 6 and (b) 7 NP OM arrays obtained with electrodynamic coupling disabled. **(c-d)** Near-field intensity for (c) 6 and (d) 7 NP OM arrays obtained with electrodynamic coupling disabled. **(e-f)** Simulated coherent images for (e) 6 and (f) 7 NP OM arrays obtained with electrodynamic coupling enabled. **(g-h)** Near-field intensity for (g) 6 and (h) 7 NP OM arrays obtained with electrodynamic coupling enabled.

4 Imaging theory

In this section we derive expressions for the electric field intensity in the near-field and the image plane presented in the main text. In the near-field of a single particle, the electric field, E_0, is due to an incident field and a scattered field

$$E(\rho, \phi) = E_0 + \tilde{A}\frac{e^{ik\rho}}{k\rho} \tag{S2}$$

where $\tilde{A}$ is a complex number (constant) related to the NP's polarizability. The intensity is then

$$I(\rho, \phi) = E_0^2 + 2E_0|\tilde{A}|\frac{\cos(k\rho + \varphi_s)}{k\rho} \tag{S3}$$

where φ_s is a phase shift factor. For a point dipole $\boldsymbol{\mu}_i$ located at the origin, the field in the image plane is

$$\boldsymbol{E}(\rho, \phi) = \frac{\omega^2}{\varepsilon_0 c^2}\overleftrightarrow{\boldsymbol{G}}_{\mathrm{psf}}(\rho, \phi) \cdot \boldsymbol{\mu} \tag{S4}$$

where $\overleftrightarrow{\boldsymbol{G}}_{\mathrm{psf}}$ is the dyadic point-spread function[5]. In the paraxial limit, the field in the image plane is

$$\boldsymbol{E}(\rho, \phi) = \tilde{B}\frac{J_1[k\rho\sin(\theta_{\mathrm{obj}})]}{k\rho}\boldsymbol{\mu} \tag{S5}$$

where $\tilde{B}$ is a complex constant and J_1 is a Bessel function. Replacing the Bessel function by its asymptotic form,

$$J_1[x] \approx -\sqrt{\frac{2}{\pi x}}\cos(x + \pi/4) \tag{S6}$$

allows approximating the electric field as

$$\boldsymbol{E}(\rho, \phi) \propto \tilde{B}\frac{\cos(k\rho\sin(\theta_{\mathrm{obj}}) + \pi/4)}{(k\rho)^{3/2}}\boldsymbol{\mu} \tag{S7}$$

If we include the field reflected off the water-glass interface, the far-field becomes

$$\boldsymbol{E}(\rho, \phi) \propto \boldsymbol{E}_r + \tilde{B}\frac{\cos(k\rho\sin(\theta_{\mathrm{obj}}) + \pi/4)}{(k\rho)^{3/2}}\boldsymbol{\mu} \tag{S8}$$

and the intensity is

$$I(\rho, \phi) = |\boldsymbol{E}(\rho, \phi)|^2 \propto E_r^2 + 2Re(\boldsymbol{E}_r \cdot \tilde{B}^*\boldsymbol{\mu})\frac{\cos(k\rho\sin(\theta_{\mathrm{obj}}) + \pi/4)}{(k\rho)^{3/2}} + O\left(\frac{1}{(k\rho)^3}\right) \tag{S9}$$

Equation (S9) can be compared to Equation (S3), although the intensity modulation in the image falls off faster, as $\rho^{3/2}$, and has a permanent, fixed phase shift of $\pi/4$ that is independent of the particles' polarizability. The fields in the image plane mirror, to a degree, the near-field of the single particle. Furthermore, the length scale in image space is given by $1/k\sin(\theta_{\text{obj}})$, which is generally longer than the $1/k$ length scale of the near-field intensity. For a perfect objective ($\theta_{\text{obj}} = \pi/2$), the length scales are identical.

5 Far-field scattering profiles

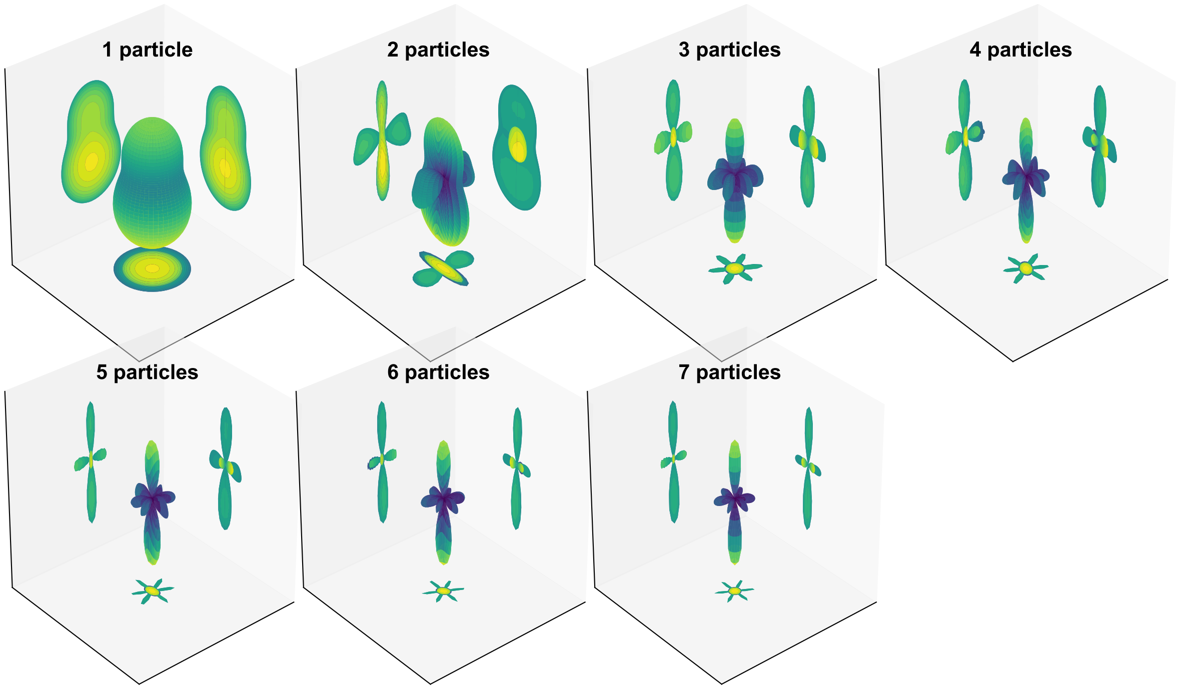

Figure S5: Far-field angular scattering of NP arrays consisting of 1-7 150 nm dia. Ag NPs at $\lambda = 800$ nm and interparticle spacing of 600 nm in a horizontal (x, y) plane. Projections of the far-field are shown along each Cartesian plane. 6 lateral scattering lobes emerge once the array has 3 or more particles. The incident field propagates along $+\mathbf{z}$; i.e., in the upward direction in each panel.

Figure S5 shows isometric views of the 3D far-field scattering profiles of OM arrays consisting of 1-7 particles obtained from GMMT simulations. The scattering profiles are also projected onto the x-y (bottom), x-z (left), and y-z (right) coordinate planes. The incident field is circularly polarized and propagates in the +z direction (upward). These 3D representations should be compared with the projections shown in main text Figure 3. While the scatting profile of a single particle is cylindrically symmetric about the z axis, the far-field scattering of two particles becomes more complicated due to interference. Starting with three particles (forming an equilateral triangle in the x-y plane), the far-field scattering develops a 6-fold symmetric lobed structure. As more particles are added to the OM array, the lobes become increasingly sharp. The far-field scattering profiles reflect the underlying trigonal symmetry of the OM arrays that forms with circularly polarized light.

6 Collection of backscattered spectra

The trapping laser beam was reflected off a dichroic beamsplitter situated below the back aperture of a microscope objective (Nikon, 60x Plan APO IR water immersion objective, NA=1.27) in order to simultaneously achieve optical trapping and measure back-scattered spectra of OM arrays. The broadband illumination for spectroscopic studies was provided by a pulsed supercontinuum fiber laser (Fianium, WL400-4-PP), operating at maximum intensity with a 5.00 MHz pulse repetition rate, coupled to a computer-controlled variable interference filter (Fianium, SuperChrome) set to its maximum bandwidth. The output was directed by a series of mirrors towards the dichroic mirror shown in main text Figure 1 to allow the broadband beam to travel collinearly with the Ti:Sapphire laser beam. The broadband beam was focused by the microscope objective to a spot that is larger than the optically trapped nanoparticle array, ensuring that the array is fully illuminated. In addition to the broadband beam, the sample was illuminated by a 470nm blue LED (Thorlabs, M470L3) transmitting through a dark-field condenser.

The back-scattered light (and the darkfield scattering of the 470nm LED illumination) was collected by the same objective and passed (transmitted) through the dichroic beam-splitter, as well as a notch filter (Semrock StopLine NF03-785E-25) to remove the back-scattered trapping laser light. The back-scattered light was split 80:20; 20% of the light was directed towards a sCMOS camera for imaging (Andor, NEO-S-S-CL3). A dual channel imaging system (Optical Insights, DualView) was used to spatially separate the dark-field images from the back-scattered laser light on the sCMOS array detector.

The remaining 80% of light was directed towards a spectrograph (Andor Shamrock SR-193i-B1-SIL). To ensure the trapping laser light was filtered out, a second notch filter (Chroma, ZET785NF) was placed in the beam path between the microscope and the spectrograph. A pair of relay lenses (Thorlabs AC508-100-B-ML) with focal length, f=100mm, were then used to bring the resulting spectrum from the spectrograph to a second sCMOS detector (Andor, NEO-5-5-CL3). Both sCMOS detectors were synchronized so that the spectral measurement would be taken at the same time as the images of the OM arrays. Once an OM array had formed, the acquisition cycle of both detectors were started and 1000 images and spectra were acquired at a rate of 160 Hz (frames per second).

Frames in the videos with well-ordered OM arrays were detected using our lattice fitting procedure[4]. Spectral images corresponding to frames with well-ordered OM arrays were separated out, Gaussian

smoothed using ImageJ, and then averaged together into one spectrum. The background spectra, obtained with no particles present, were also averaged into one spectrum in the same manner. After subtracting the background, the counts across a vertical strip of nine pixels centered around the maximum of each column in the spectrum were then averaged together to give the scattering spectrum of each particular size nanoparticle array. After this procedure was performed for nanoparticle arrays with 1-7 particles, the spectra for arrays with 2-7 particles were divided by the single particle case to give the normalized scattering spectra. The final results are shown in main text Figure 4.

7 Evaluation of induced-polarization

The induced-polarization of the ith particle p_i (or $p_{on,i}$) of a given OM structure can be calculated by taking the 2-norm of the electric dipole of the ith particle within the OM array (structure). The 2-norm of a vector is the square root of the sum of squares of all the entries in the vector. We also calculate the 2-norm of the electric dipole of that particle at the same position but with all the other particles absent, denoted as p_0. Since the system is a plane wave electromagnetic field incident on the OM, p_0 is the same for all particles with identical sizes and permittivity. The average induced-polarization of the OM array is obtained by $p_{avg} = \sum p_i/N$, where N is the number of particles. The average enhancement of the induced-polarization of the OM array is p_{avg}/p_0.

The coupling of the particle-particle scattered fields can be turned on and off in the simulation. When the coupling is turned off, the induced-polarization of each particle, $p_{off,i}$ is only due to the incident field. With both $p_{on,i}$ and $p_{off,i}$ defined, the induced-polarization enhancement of each particle is defined as: $\left(p_{on,i} - p_{off,i}\right)/p_{off,i}$.

8 Phase of induced-polarization in large NP arrays

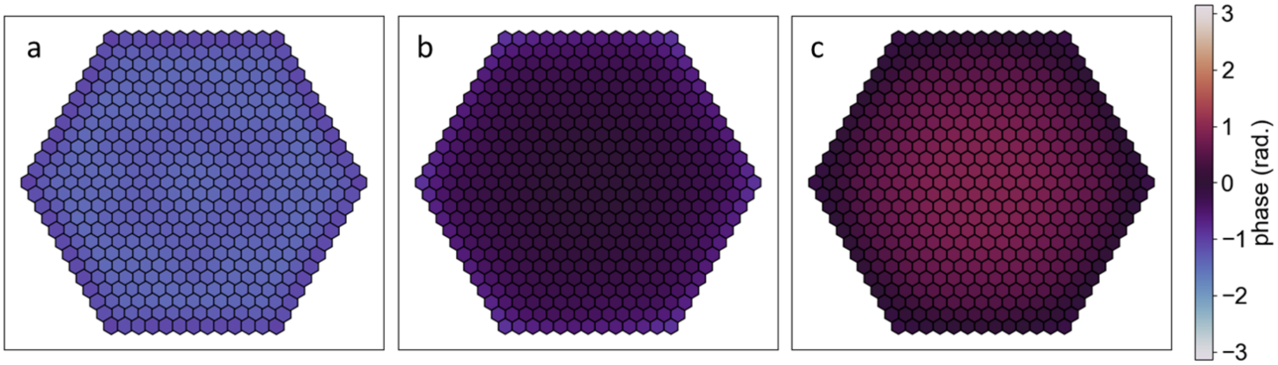

Figure S6: Phase of the polarization of each particle in a 469 NP array with variable lattice constant and illuminated by circularly polarized plane-wave of 800 nm wavelength. **(a)** 600 nm lattice constant. **(b)** 670 nm lattice constant. **(c)** 680 nm lattice constant.

Figure S6 shows a visualization of the phase of the induced-polarization of each particle in a 469 NP trigonal lattice array illuminated by a circularly polarized $\lambda = 800$ nm plane-wave in a water medium ($n = 1.33$) and for lattice constants of (a) 600 nm, (b) 670 nm, and (c) 680 nm. When the lattice spacing is 600 nm it is nearly equal to the wavelength of the incident light in the water medium. In that case the phase of each particle's induced-polarization lags behind that of the incident field by approximately $-\frac{\pi}{2}$ radians. At the lattice spacing where scattering by the array is maximized, 670 nm, the particles in the center of the array are in-phase with the incident field, and the phase of the particle polarization becomes progressively more delayed moving outward, reaching a delay of approximately $-\frac{\pi}{4}$ radians in the outermost layer. At a lattice spacing of 680 nm, the phase of the particles near the center of the array is advanced by approximately $\frac{\pi}{4}$ compared to the phase of the incident field, and becomes closer to the phase of the incident field moving outward toward the array's periphery. The strong dependence of the phase of the particles' induced-polarization in the array on the lattice spacing demonstrates that even away from the surface lattice resonance the excitation of the array is collective in nature.

9 Comparing GMMT calculations using MiePy with Finite Difference Time Domain (FDTD)

The GMMT calculations performed here are based on a code named MiePy found on Github[6]. The following complements details provided in the main text. First, we determined that only dipolar and quadrupolar terms in a multipolar expansion of nanoparticle modes and interactions are necessary for accurate simulations of optically bound (100-200nm dia.) Ag and Au nanoparticles at typical optical binding separations (e.g., 600nm).

Figure 6.1 from J. A. Parker's Ph. D. thesis[7] compares the runtime performance and accuracy of GMMT and the well-known finite difference time domain (FDTD) method on the same computer for an optical matter array consisting of N particles, where N = 1; 2; 3; 4; 5; 6; 7. We are able to calculate one-million time-steps (a roughly 1 second trajectory) in 20 minutes of CPU time using GMMT. FDTD takes about 700 days (estimated) to do the same computation. Although FDTD can be parallelized or run on a GPU, it would require significant computational resources to be competitive with GMMT.

Even worse for FDTD, for these spherical sub-wavelength particles it proves to be less accurate than GMMT for all grid sizes considered. The relative error in the forces on the particles is seen to approach zero as the FDTD grid spacing (Δx) is reduced. Unfortunately for FDTD, the runtime performance scales as $O(\Delta x^4)$ and obtaining accurate results will take an extraordinarily long time.

For these reasons, GMMT is generally a superior method for simulating optical matter. It should be noted that GMMT is more niche than FDTD, and consequently there are few available codes for running GMMT while there are many available codes (open-source and commercial) for running FDTD. Therefore, an open-source GMMT software, MiePy, was developed.

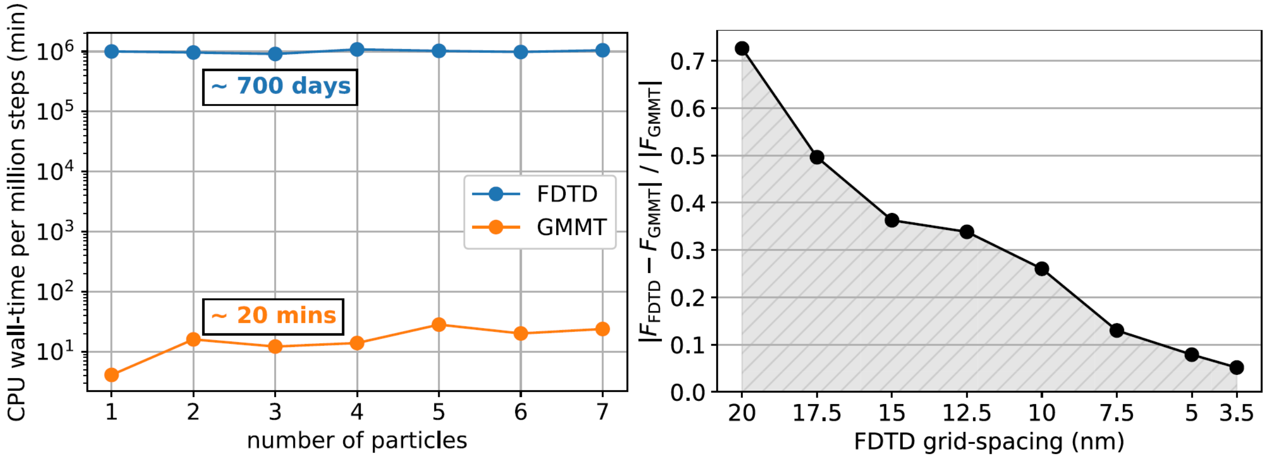

Figure S7: Computational performance and accuracy of the generalized multiparticle Mie theory (GMMT) compared to the finite-difference time-domain (FDTD) method. **(left)** Wall-times for GMMT and FDTD simulation methods for 1 - 7 particles (using a single CPU processor; AMD Ryzen 7 2700X). GMMT is 4 - 5 orders of magnitude faster. **(right)** Relative error between the forces in FDTD and GMMT for the 7 particle case. As the grid-spacing is lowered in FDTD, the forces converge to the values of $\boldsymbol{F}$ in GMMT, suggesting that GMMT is more accurate than FDTD. Figure reproduced with permission from ref 7. Copyright 2020 John A. Parker.

References

[1] Crocker, J. C.; Grier, D. G. Methods of Digital Video Microscopy for Colloidal Studies. *J. Colloid Interface Sci.* **1996**, *179*, 298–310.

[2] Sbalzarini, I. F.; Koumoutsakos, P. Feature Point Tracking and Trajectory Analysis for Video Imaging in Cell Biology. *J. Struct. Biol* **2005**, *151*, 182–195.

[3] Shivanandan, A.; Radenovic, A.; Sbalzarini, I. F. MosaicIA: an ImageJ/Fiji plugin for spatial pattern and interaction analysis. *BMC Bioinformatics* **2013**, *14*, 1–10.

[4] Chen, S.; Peterson, C. W.; Parker, J. A.; Rice, S. A.; Ferguson, A. L.; Scherer, N. F. Data-Driven Reaction Coordinate Discovery in Overdamped and Non-Conservative Systems: Application to Optical Matter Structural Isomerization. *Nat. Commun* **2021**, *12*, 1–11.

[5] Novotny, L.; Hecht, B. *Principles of Nano-Optics*; Cambridge University Press, 2012.

[6] https://github.com/johnaparker/miepy.

[7] Parker, J. A. Collective Electrodynamic Excitations and Non-conservative Dynamics in Optical Matter and Meta-Atom Systems. Ph.D. thesis, University of Chicago, 2020.
